# Encountering a bait is necessary but insufficient to explain individual variability in vulnerability to angling in two freshwater benthivorous fish in the wild

**DOI:** 10.1371/journal.pone.0173989

**Published:** 2017-03-16

**Authors:** Christopher Thomas Monk, Robert Arlinghaus

**Affiliations:** 1 Department of Biology and Ecology of Fishes, Leibniz-Institute of Freshwater Ecology and Inland Fisheries, Berlin, Germany; 2 Division of Integrative Fisheries Management, Department of Crop and Animal Sciences, Faculty of Life Sciences, Humboldt-Universität zu Berlin, Berlin, Germany; University of Pretoria, SOUTH AFRICA

## Abstract

Fish personality traits, such as swimming activity, or personality related emergent behavioural properties, such as the degree of space use shown by an individual fish, should affect encounter rates between individual fish and fishing gear. Increased encounters should in turn drive vulnerability to capture by passively operated gears. However, empirical evidence documenting a relationship between activity-based behaviours and vulnerability to capture by passive fishing gear in the wild is limited. Using whole-lake acoustic telemetry, we first documented significant repeatabilities over several months in a suite of encounter rate-associated behaviours (swimming distance, activity space size, time on baited feeding sites, switching frequency among baited feeding sites, distance to the lake bottom) in two recreationally important benthivorous cyprinid species, the common carp (*Cyprinus carpio*) and tench (*Tinca tinca*). We then experimentally targeted both species using stationary angling on baited feeding sites. Individual fish regularly visited the angling sites, documenting that the fishes encountered the angling baits. When attempting to explain individual variation in vulnerability as a function of repeatable behavioural traits, we found no evidence of a significant relationship among various encounter-based behaviours and vulnerability to angling for both species. There was also no evidence for size selection or for energetically less conditioned fish to be more vulnerable. The data cumulatively suggest that fine-scale behaviours after encountering a bait (e.g., frequency of bait intake) may be ultimately decisive for determining vulnerability to angling in benthivorous fish. Based on our work, fishing-induced selection on encounter-based behaviours in recreational angling for benthivorous fish in the wild appears unlikely.

## Introduction

Individual fish systematically vary in their intrinsic vulnerability to capture in a fishery [1–4]. Previous work has shown capture probability is related to a suite of morphological (e.g., size), life-history (e.g., growth rate), physiological (e.g., metabolic rate) and behavioural traits (e.g., activity) of the fish [5–7]. While our understanding of the relationship between morphological and selected life-history traits and vulnerability to different fishing gears is well advanced [8–11] other traits have received far less attention [5]. In particular, there is a knowledge gap related to the potential for behaviour-selective fisheries [11]. Behavioural traits are involved in nearly all ecological processes, such as foraging, predator avoidance, and dispersal, thereby affecting a range of important biological outcomes such as energy turnover, mortality, spawning and nutrient cycling [12]. Hence, fisheries-induced changes in the behavioural repertoire within wild fish populations can have important ecological and managerial ramifications [7,13–16].

The idea that fisheries selectively capture certain behavioural traits is not new [17]. It is well known that behaviour expressed by individual fish plays a key role in exposing individuals to fishing gear [18–20]. In fisheries using passive gears such as gill-nets, traps, long-lines or angling with rod and line, the behaviour of exploited fishes is especially important [20]. While encountering certain passive gears, such as gill nets, may be sufficient to induce capture [21,22] in other passively operated systems, such as trapping or angling, the individual fish must also be motivated to enter the gear or ingest the bait. It is therefore likely that in addition to activity and space-use, other behavioural traits such as boldness (i.e., risk-taking [23]) play a fundamental role in affecting individual capture probability in trapping [24] and angling [25]. We will focus on recreational angling in this paper, which constitutes the dominant use of most freshwater fish stocks in industrialized countries and is increasing also in coastal areas [26].

Alós et al. [27] presented an individual-based model focused on encounter-based mechanisms to determine the theoretical selection differentials acting on activity and space use (home range) in angler-exploited fisheries. The authors showed angling-induced selection is expected to consistently act on activity-related traits (e.g., swimming distance or exploration rate), while selection on home range should only be consistently strong when fishing is restricted to fixed position. Consistent with these expectations, in experimental studies high swimming activity has been found to consistently relate to capture probability in gill-nets and traps across a range of species [21,22,28], and in the wild Olsen et al. [29], reported no fishing-induced selection operating on home range extension in Atlantic cod, *Gadus morhua*, fished with a variety of passive gear types in a Norwegian fjord. By contrast, in an angling context, Alós et al., [30] reported negative selection differentials acting on both home range extension and swimming activity in angler-exploited pearly razor fish, *Xyrichthys novacula*, in the wild, and the correlation between swimming activity and vulnerability to angling is overall variable and inconclusive (positive finding: [31]; negative: [29,32–34] mixed evidence: [35,36]). The reasons could include methodological aspects (e.g., angling vulnerability assessments in small tanks where high swimming activity might not be required to encounter baits [37]), or species-specific responses to baits [14]. Moreover, it is likely that capture probability in angling is more complex than merely involving encountering the bait because a fish must also decide to take the bait after an encounter [20]. Hence, behavioural selection in angling may more readily relate to boldness [25] or aggression as personality traits [38] than to encounter-based behaviours per se, but more work is needed particularly under natural conditions in the wild.

For a behavioural trait to cause variation in angling vulnerability among individuals, and hence opportunity for selection, some consistent structure in the among- individual variation of that trait within the population is imperative, i.e., the trait must be repeatable among individuals [29]. There is growing awareness that such behavioural structure, usually referred to as personality or behavioural type, is frequently present in fish populations as in animal populations in general [19,39,40]. Although there are few investigations of fish personality in the wild, a handful report significant repeatability for a range of activity related behaviours across several species [29,41–44]. Recent advances in technologies such as three-dimensional high-resolution acoustic telemetry now allow measurement of the behaviour of unique individuals at a relatively fine scale over months to years in natural ecosystems [45,46]. Collecting positional data in this way is a useful tool for measuring encounter rates and associated movements and spatial behaviours in the wild, thereby adding realism to support (or refute) the hypothesis that activity and other types of encounter-based behaviours are systematically related to vulnerability to angling. Furthermore, with such long term data, the consistency of behavioural types can now be estimated under realistic conditions in the wild [42].

An additional challenge in teasing apart the relationship between behaviour and angling vulnerability is behavioural traits can be linked and confounded with other traits on which fishing selection can also act, particularly body size [5,38,47,48]. Repeatable behaviours can also correlate with age, growth rate, fecundity, morphology or metabolism [49,50]. Positive size selectivity is common feature in recreational angling [51] and there are several examples in which fast growing fish [31,52–54], as well as fish with high metabolism [48] are harvested preferentially by anglers. Therefore, approaching a complete understanding of angling vulnerability's causes requires measurement of multiple traits in addition to behavioural ones.

The study objective was to experimentally test in the wild whether behaviours associated with bait encounter rates increase vulnerability to capture in hook-and-line recreational angling using two benthivorous fishes (common carp, *Cyprinus carpio*, and tench, *Tinca tinca*) commonly targeted by coarse (i.e., non salmonid) anglers in European freshwaters [55,56] as model species. To that end, we first evaluated individual differences in a suite of behaviours over four months in an experimental lake using high-resolution acoustic telemetry in response to baited feeding sites used commonly in angling for benthivorous fishes [57]. Second, during the last two months of the same study period we angled for the tagged fishes in a controlled manner to measure the relationship between the individual behaviours and the capture rate of the fish. We controlled for the effects of size as a morphological trait and lipid content as a measure of energetic state to understand the importance of behavioural effects relative to morphological and energetic traits/status. We simultaneously tracked two benthivorous cyprinid species to understand whether drivers of vulnerability were similar among species with a similar foraging niche. We hypothesized that 1) consistent individual differences in personality-related behaviours are present for both carp and tench in the wild and 2) individual carp and tench that encounter baits more frequently are captured earlier and more often than fishes with opposing behavioural phenotypes. Finally, we hypothesized that 3) larger fish [51] and fish with a lower lipid content are more vulnerable to angling because lower conditioned fish are known to take more risks during foraging [58].

## Methods

### Ethics statement

The invasive animal procedures (including surgeries and recaptures) were ethically approved by the responsible State Animal Welfare and Animal Experimentation Agency (Landesamt für Umwelt, Gesundheit und Verbraucherschutz) in Brandenburg, Germany (project reference 2347-21-2014) according to the German Animal Protection Act. Our study did not involve any endangered or protected species, and no animals were sacrificed. Surgical implantation of the acoustic telemetry tags was conducted after fish were anaesthetized by a 9:1 95% EtOH:clove-oil solution, and all efforts were made to minimize handling and harm. After release of the fish into the study lake, we observed 15 individual carp with infected surgical wounds and found one additional dead carp with an unknown cause of death. Wounds were judged as likely non-lethal and carp were thus not treated as treatment was considered to cause additional stress to the fish. Some of the wounded fish were recaptured later with fully healed wounds.

### Study site

Kleiner Döllnsee is a 25 ha weakly eutrophic, lake (for limnological details see [46]) in northern Brandenburg, Germany (52°59'32.1''N, 13°34'46.5''E). The shallow, 7.8 m deep (4.4 m average depth) lake is surrounded by reed (*Phragmites australis*). When the lake stratifies between May and October, the water below c. 4 m depth is anoxic. Only experimental fish sampling, mainly targeting top predators such as pike, *Esox lucius*, e.g. [59], has taken place at Kleiner Döllnsee since 1992. The Leibniz Institute of Freshwater Ecology and Inland Fisheries has been the fishing rights owner of Kleiner Döllnsee since 1992, and in this position has full permission to conduct the study on the premises. The lake naturally hosts tench [60], but at the time of the study no carp were present in the lake due to a winter kill off of the original stock.

### Telemetry system

In 2009, the lake was equipped with the high-resolution whole lake acoustic telemetry system (Lotek Wireless, Newmarket, Canada) (see [46] for extensive details), used to record three-dimensional positions of tagged fish. The system comprises 20 receivers positioned two metres underwater, distributed throughout the lake. Each receiver stores the arrival time from ultrasonic signals arriving from transmitters surgically implanted into the body cavities of individual fish or from beacon tags, and depending on the tag, the water temperature and depth of the tagged fish was also recorded. After manually downloading data, each fish's positions can be calculated from the disparity in signal arrival time at various receivers using proprietary software from the supplier as detailed in [46]. Positioning errors are strongly minimized via post-filtering involving a Hidden Markov Model (see [46] for details). Careful performance studies have been conducted and published elsewhere [46], but briefly, the system is able to detect tagged individuals with a very high probability in the sublittoral and open water body. Detection probability increases in open water, whereas fish in the reed belt of the lake are rarely detected. The average system accuracy ranges from one to five metres, depending on environmental conditions, habitat and season [46]. Macrophytes are known to reduce data yield, but during the study year there were very few submerged macrophytes. It is therefore reasonable that detections were extremely probable from sublittoral habitats and poor or absent from highly structured littoral habitats (i.e. reed).

### Fish collection and transmitter implementation

#### Carp

All carp were hatchery born and lived their entire lives in earthen ponds surviving on natural food supplemented by formulated feed. In June 12–13, 2015, 91 carp (total length: 40.6–72.2 cm; wet weight: 945–6934 g) were seined from three 12 x 5 m ponds and held in oxygenated fibreglass tanks. Carp were anaesthetized individually in a 100 L opaque container using a 9:1 95% EtOH:clove oil solution (Carl Roth, Karlsruhe, Germany) added at 1mL L^-1^ and the total length (TL) and wet mass were measured. Fish were also photographed for later identification in case of tag loss. We took triplicate fat content readings in the epaxial muscle immediately above the lateral line on both sides of each carp using a calibrated FFM-992 non-lethal and non-invasive microwave fat meter (Distell Inc., West Lothian, Scotland). Measuring lipid content in this way is known to correlate closely with whole-body energy density in carp [61].

Acoustic telemetry transmitters (model MM-M-TP-16-50, transmission frequency: 5 s, dimension: 16 by 85 mm, wet weight: 21 g; Lotek Wireless, Canada) were surgically implanted into the fish according to procedures outlined elsewhere [39,62]. All transmitters were equipped with a pressure sensor to record its depth and a temperature sensor which records water temperature once per minute alternating with water depth. Passive integrated transponder (PIT) tags (23 mm length, 2 mm width, Oregon RFID, OR, USA) were inserted through the incision, similar to Skov et al., [63], for identification upon capture by angling. All surgical tools and acoustic telemetry tags were sterilized with a mixture of tap-water and 7.5% povidone-iodine (PVP; Braunol^®^; B. Braun, Kronberg, Germany) before each surgery. Surgeries lasted an average of 6:15 minutes (standard deviation (sd) = 1:39 minutes), and each fish received 4–5 sutures using PDS-II adsorbable monofilament suture material and FS-1 3–0 needles (Ethicon, USA). Following surgery, fish were immediately placed in an aerated transportation tank, and after all surgeries the fish were transported and released into Kleiner Döllnsee (water temperature: 23.6°C). On September 5, acoustic transmitters of the same specifications were implanted into an additional 24 carp (TL: 43.0–70.7 cm; wet mass: 1117–5872 g, surgery time mean ± sd: 6:29 ± 1:39 minutes). New fish were tagged to counter the observed significant tag loss, known to be a prevalent problem in carp tagging [64,65]. The newly tagged fish were also released into Kleiner Döllnsee (water temperature: 18°C) for behavioural measurements and angling vulnerability assessments.

#### Tench

Between July 17 and September 17, 2015, 36 tench (TL: 37.1–52.3 cm, wet mass: 736–2099 g) were sampled, tagged and released into Kleiner Döllnsee (water temperature: 17–25°C). The tench came from three sources with different capture methods: Kleiner Döllnsee (n = 5; angling), Groß Vätersee (n = 9; gillnets), a small nearby research lake of similar trophic state and the River Oder (n = 22; gillnets and fyke nets) where tench were purchased from a commercial fisher. Capturing fish by different gears was meant to increase behavioural variation because all gears are expected to be somewhat behaviourally selective. Sampling tench was similar to carp; however, the fat meter was unavailable. Surgeries to insert acoustic transmitters (MM-M-TP-11-28; transmission frequency: 35 s, dimension: 12 by 65 mm, wet weight: 6.5 g; Lotek Wireless, Canada) and PIT tags lasted on average 6:38 minutes (sd = 1:43 minutes), and fish received 3–4 sutures. Transmitters were also equipped with a pressure and temperature sensor.

### Experimental timeline

**Fig 1:**
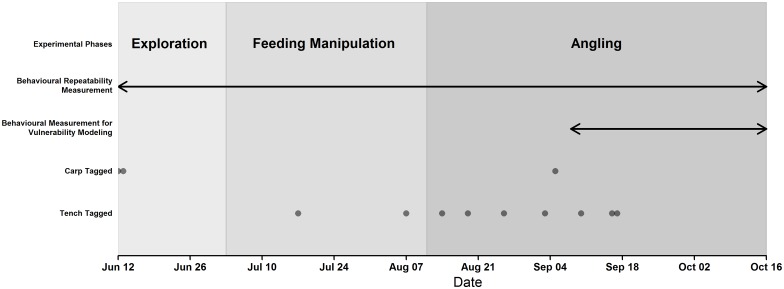
Experimental timeline. A timeline of the experiment's three phases, the periods where daily behavioural measurements were considered for repeatability calculations and for behavioural models of vulnerability and the dates where carp and tench were tagged and added to the dataset. Note that behaviours of tench tagged after September 8 (n = 3) were not included in the behavioural models of vulnerability to angling.

The stages of the study from release, observation of exploration of a novel environment to angling exploitation are summarised in Fig 1. We tracked fish from first stocking, June 12, until October 15. This period was divided into three experimental phases: initial exploration (June 12—July 2), feeding manipulation (July 3—August 11) and angling (August 12—October 15). During initial exploration fish swam freely in the lake, during feeding manipulation eight littoral sites in the lake were baited differently each week simulating pre-baiting common in carp and tench angling, and during the angling phase we angled for the fishes on four baited sites. The purpose of the exploration and feeding manipulation phases was twofold: 1) as an acclimation to the new environment and pre-baited patches required later for angling and 2) as novel contexts for repeatability calculations as animal personality is defined as consistent context independent among-individual variation in behaviour [66]. In the angling phase we recorded which fish were vulnerable to capture by standardized angling gear.

A range of behaviours (see below) of the fishes were estimated throughout the experiment. We considered every day in our repeatability analysis, excluding 19 non-consecutive days where downloading data from the telemetry system prevented positioning. However, in our vulnerability models we only considered behaviours from September 8 until October 15 where the greatest number of fish generated positions simultaneously, thus ensuring behavioural scores of all fish in the analysis were directly comparable and derived from the same environment. Shorter-term behaviours could be considered representative of longer-term "personality" if the behaviours were found to be repeatable over the duration of the experiment, which we explicitly tested *a priori*.

### Feeding site development and manipulation

**Fig 2:**
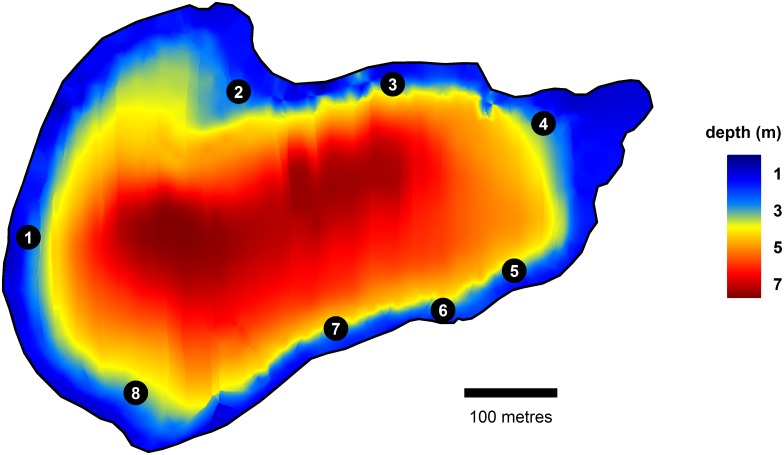
Kleiner Döllnsee depth map and feeding site locations. The depth map of Kleiner Döllnsee and the locations of the eight feeding sites in the lake. The radius of each of the circles that represents the feeding sites is scaled to 15 m, which is the distance at which a fish was considered present at the feeding site. Sites 2,4,6 and 8 were angled.

We defined eight GPS-marked feeding sites (Fig 2) along the shoreline and used them to deliver corn (Ruthild, Futtermittelhandel, Templin, Germany) as is typical in carp and tench angling [56,57,67]. The number of feeding sites was varied weekly to expose the fish to various ecological contexts (Table 1). Sites were distributed around the lake, but chosen so at least four were accessible for angling from a fixed position on the bank, and all would be in 3.5–4 m depth to avoid possible hypoxic conditions in deeper water.

**Table 1:** Sequence of feeding manipulations to modify the context in the lake for repeatability measurements.

Week	Treatment	Context	Sites used (see Fig 2)
July 3—July 13	Food added to one feeding site	Novel food source	2
July 14—July 20	Food added to one feeding site	Scarce competitive food source	2
July 21—July 27	Food added to eight feeding sites	Predictable abundant food source	1 to 8
July 28—August 3	Food added to four feeding sites	Unpredictable food source	2, 4, 6 and 8
August 4—August 7	Food added to four feeding sites + enclosed pike beside two feeding sites	Risk at food source	2, 4, 6 and 8

On the feeding sites, soaked, cooked corn (2 kg) was delivered to the lake's bottom in mesh bags (similar to [42,68]) twice daily per site. Feeding twice per day was continued for four days during a weekly feed treatment. On the fourth day's afternoon feeding the feed amount was increased to 14 kg per site, to maintain the interest of the carp at the site over the next three days (a weekend period) until the next weekly treatment.

### Experimental angling

During the angling phase, we maximised effort over four days each week, (in the first angling week there were only three angling days for logistical reasons). Angling occurred during daylight (1,299 rod hours) and nighttime (843 rod hours). In total the lake was fished 85.7 rod hours ha^-1^; a moderate carp fishing intensity typical of German carp fisheries [69]. Four feeding sites (sites 2,4,6,8; in Fig 2) were chosen as angling sites, and in a given week these sites were occupied by one to four anglers. If fewer than four anglers were present, fished sites were switched randomly to distribute fishing pressure. We used standard carp fishing gear with a bolt rig leading to shallow self-hooking [70] (for details about the angling rig and techniques see S1 Appendix) and two to three rods per angler. As bait, boiled feed-corn (2–3 pieces) was used on one rod and 14 mm diameter boilies [57] (M&M Baits Neuenkirchen-Vörden, Germany) on the other. Boilies were also added to the feed sites as pre-bait during the angling phase (1 kg per feeding). The species, TL, bait used and capture time were recorded for all captured fish. Captured carp and tench were weighed and identified by PIT tag or photograph (by matching scale pattern [71]). Additionally, by-catch was common (*Abramis brama* in particular), which is not reported in this paper.

### Daily behavioural metrics

A suite of behaviours related to bait-encounter, movement, space use and feeding site use were estimated daily for each individual. Behaviours considered were 1) distance swam, 2) average distance from the lake bottom, 3) daily activity space size, 4) total time in the structure-free open sublittoral area, 5) total time at the feeding sites and 6) the number of switches among feeding sites. All measurements represent behaviour outside of shelter (macrophytes and reeds) as the telemetry system functions best in open water [72]. For detailed behavioural calculations see S1 Appendix.

The distance swam and the activity space size were measured because a greater distance swam and a larger activity space should result in a higher probability of encountering fixed baits [21,27,73]. We measured distance to the lake bottom because increased time near the lake bottom may indicate increased foraging for benthic prey and subsequent bait encounters as carp and tench are benthivores and the bait lies on the lake bottom. Furthermore, we assumed increased time in the structure-free sublittoral zone indicates reduced time in refuge and also higher encounter rates with bait. Finally, we measured behaviours directly related to bait encounters on feeding sites. Time spent on feeding sites directly measures potential encounter rate; however, as all feeding sites were not constantly angled, a fish switching feeding sites more frequently should also encounter baited hooks more frequently. Note, during the shorter period of behavioural measurement used in the vulnerability models food was only introduced at the angled feeding sites and therefore the possibility that a fish is spending time at a feeding site where angling is not occurring was excluded. Also, through the entire study time spent at feeding sites where food was added strongly correlated with time spent at the subset of feeding sites which were eventually angled (see S1 Appendix). Therefore long term repeatability for time spent at all feeding sites was representative of behaviour expressed on angling sites in autumn.

For models assessing the relationship between behaviour and angling vulnerability, and for a comparison of carp and tench behaviours, individual fish were characterized by aggregations of daily behaviour. The distance to the bottom of the lake was described by the arithmetic mean, while all other behaviours were summarised by geometric means because of left-skewed distributions of daily behaviour. To log (natural) transform the data for the geometric mean, we replaced zeros with very small values (five orders of magnitude below the maximum behavioural score).

### Statistical analysis

#### Sample sizes and tag loss

Reliability that positions were generated from live fish was verified through visualizations of daily positions for each individual over the study period. It was easy to identify living individuals because both carp and tench showed little evidence of long-term (several days) stationary behaviour and instead readily moved around the lake (see S1 Appendix). Predation was highly unlikely given the size of fishes we used.

We observed high tag loss with only 33 out of 114 carp producing valid data for analysis of the relationship between behaviour and vulnerability over several months (see S1 Appendix for a summary of detections by the telemetry system for these carp). Sample size was larger for repeatability calculations (N = 58 individual carp). Significant tagging mortality was unlikely as we recaptured many fish that had lost their tags alive. Most transmitter loss can be qualitatively attributed to sepsis of the surgical incision observed in a number of recaptured carp. Many recaptured carp were found with a slightly open and infected wound, and these fishes were later identified as lacking positional data soon after tagging. Other recaptured carp which retained their tags appeared healthy with healed incisions. Substantial tag loss has been observed in carp before [64,65]. In tench, 28 of 36 individuals produced valid data for the vulnerability models, but only 25 individuals were used in the analysis of the relationship between behaviour and vulnerability because three tench were added near the end of the fishing period (see S1 Appendix for a summary of detections by the telemetry system for tench). In the repeatability calculations behavioural data were included from 33 individual tench. The cause of tench without valid positional data is unknown as no sepsis was observed on any surgical wounds for tench, therefore tag malfunction is most likely.

#### Between species behavioural comparison

We compared carp and tench behaviours to understand population level species-specific behavioural differences. We restricted this comparison to the period between September 8 and October 15, when the largest number of individuals were simultaneously generating data. We used Mann-Whitney-U tests to compare the distributions and medians of behaviours between carp and tench, because of the non-Gaussian distribution of the behavioural data. Multiple comparisons were corrected for with the Holm-Bonferroni p-value adjustment.

#### Repeatability assessment

We estimated repeatability for all carp and tench daily behavioural measures. Within- and between-individual covariances of a behaviour were partitioned across days using univariate mixing models fit with Markov Chain Monte Carlo procedures [74]. Each model was fit with fish identity as random intercepts and run for 500,000 chains with a burn-in of 1000, and every 100 chains were sampled to prevent autocorrelation. Poisson models with additive overdispersion were fit for all behaviours except distance to the lake bottom, which was fit with a Gaussian error distribution. We used uninformative priors appropriate for each error distribution [75] and calculated repeatability using the appropriate equation found in [76]. Each model's goodness of fit was evaluated by examination of the trace plots (See S2 and S3 Appendices). We ran each model five times to ensure consistent estimates (results of only one model per behaviour are reported).

#### Assessment of catch rates over time

We checked for temporal changes in vulnerability that may indicate hook avoidance or relate to temporal behavioural changes. To that end, we predicted catches in response to cumulative fishing days from the onset of angling (August 12 to October 15) using a generalized linear model with a log link and Poisson error offset by daily rod-hours as per [77].

#### Modeling vulnerability to angling

To test for the behavioural, morphological and energetic influence on the speed of capture from the onset of angling a Cox-proportional hazards model was used. As additional fish were stocked partway through the experiment, "counting" censoring was used [78]. Capture was scored as a binary variable (1 = capture, 0 = no capture). Start time was from the beginning of the fishing period for fish added in June, the stocking time for the fish added in September 5, and immediately after a capture for recaptured fish. Time was in units of fishing effort rather than real time, so periods without fishing remained unconsidered. Additionally, a frailty term for repeated measures was added to the model to account for recaptured individuals.

A logistic regression was used to test for the relationship between behaviour and capture probability. In this model recaptures were not considered. Carp and tench were modelled separately because their behavioural scores were too different to combine into one model. We considered all behaviours, TL and lipid percentage as predictors of carp capture probability, but not lipid percentage in tench models because of lack of measurement. Variance inflation factors (VIF) were calculated to avoid variable collinearity. Any variables with a VIF above three were excluded from models [79]. In carp, TL, lipid percentage, time at the feeding sites, activity space size and distance to the bottom of the lake were included in the final models and in tench, TL, number of switches among feeding sites, distance swam and distance to the lake bottom. All predictor variables were standardized by z-transformation.

The best models were selected using multimodel inference [80] using conditional Akaike Information Criterion (AICc) for small sample sizes and parameters from models with an AICc score less than two were averaged. Given low sample sizes, a power analysis (alpha = 0.05, beta = 0.8) was conducted for a single continuous variable logistic regression using the estimated standardized effect sizes for each variable included in the best model, when all other variables were held at their mean. The power analysis was conducted in R using the powerMediation package. All statistical analyses were done with R version 3.2.1 (CRAN) using packages (MCMCglmm, survival, frailtypack, and MuMIn).

## Results

### Carp and tench behaviours in the wild at the population level

**Fig 3:**
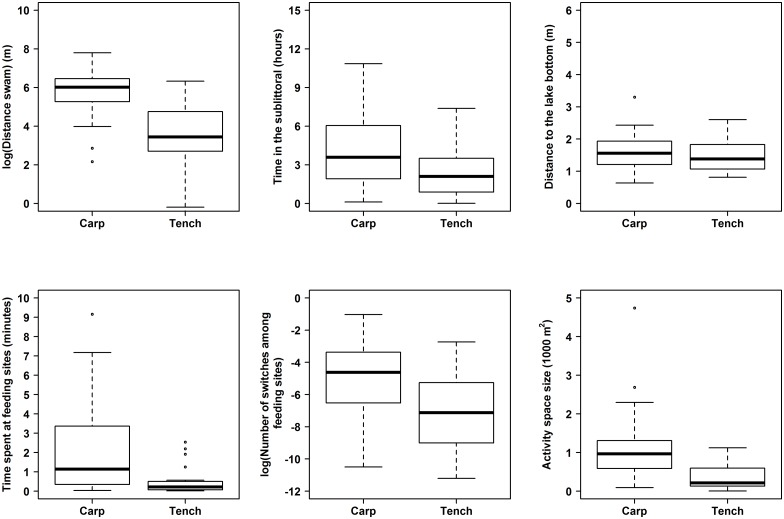
A comparison of carp and tench behaviour. Boxplots of summarised behaviours from September 8 to October 15, 2015 for six carp and tench behaviours. Distance swam and number of switches among feeding sites are on a log scale to facilitate visual comparison. The black bar represents the 50th percentile and the whiskers represent 1.5 times the interquartile range.

**Fig 4:**
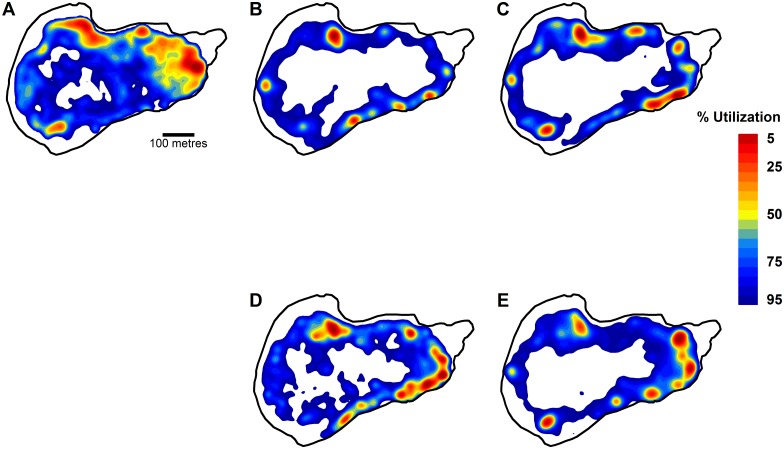
Space use of carp before feeding, and carp and tench during feeding and during angling. Kernel utilization distributions of the entire population of carp before feed sites are added to the lake (A), when feeding sites are present but before angling has begun (B) and during angling (C), and of tench when feeding sites are present but before angling has begun (D) and during angling (E). See Fig 2 for the locations of the feeding sites.

Carp (n = 33) travelled significantly further (U = 795, p < 0.001), spent more time in the sublittoral (U = 569, p = 0.026) and had a larger activity space (U = 677, p < 0.001) than the 25 tench tracked (Fig 3). Carp, also spent significantly more time at the feeding sites (U = 640, p < 0.001) and switched feeding sites more frequently during a given day (U = 627, p = 0.002) than tench (Fig 3). Only the distance from the bottom of the lake was not found to differ significantly between the two species (U = 340, p = 0.26) (Fig 3). The carp population shifted their core home range to the feeding site locations once food was added to the lake, and this shift in site use remained through the angling period (Fig 4). The tench population also had the core home range areas around the feeding sites (Fig 4) indicating that both species readily encountered the baits and strongly reacted to the addition of corn (See S1 and S2 Videos).

### Repeatability of carp and tench behaviours in the wild

**Fig 5:**
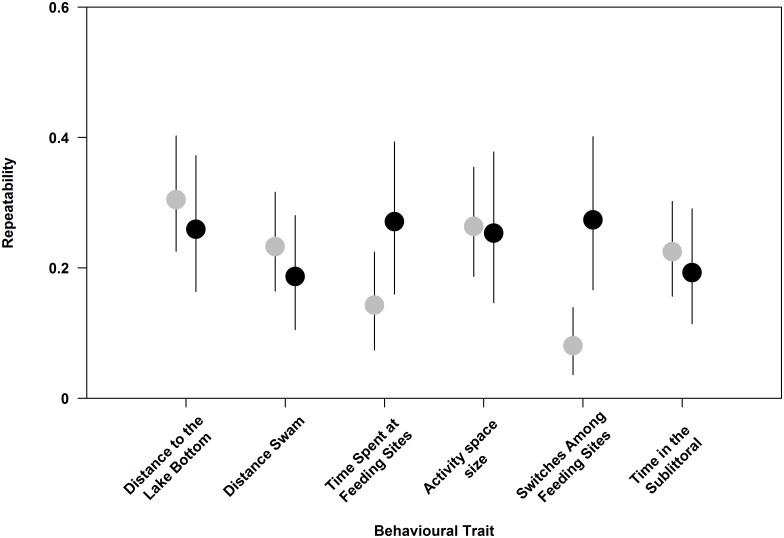
Carp and tench behavioural repeatabilities. Repeatabilities and their 95% credible intervals for six behaviours over the entire experimental period and across individual lake manipulation treatments. Grey points represent carp and black points represent tench.

All measured behaviours were significantly repeatable over four months of tracking, with mean repeatabilities ranging from a low of 0.08 to a moderate repeatability of 0.30 (Fig 5; for within- and among-individual variance estimates see S1 Appendix). The 95% credible intervals for repeatabilities ranged between 0.04 and 0.41 (Fig 5). Note, the 95% credible intervals overlapped among carp and tench; therefore the differences in repeatability of behaviours among species were not significant (Fig 5).

### Temporal development of catch data

In total, 20 carp were landed once, four twice and five carp were lost before landing for identification. Of the landed carp, nine had acoustic transmitters, two of which were captured twice. Tench were captured 32 times and one tench was known to be lost before identification. Nine of the captured tench had an acoustic transmitter, two of which were captured twice. The other landed tench were wild and not considered in this study. Including hooked fish that were not landed and measured, the catch per unit effort (CPUE) for carp was 0.018 ± 0.033 fish rod h^-1^ (mean ± sd) ranging from 0 to 0.135 fish rod h^-1^, while the CPUE for tench was 0.016 ± 0.03 fish rod h^-1^ (mean ± sd) ranging from 0 to 0.182 fish rod h^-1^. The CPUE of tench and carp over the entire angling period was very similar with one carp and tench captured every 32 hours when fishing with two rods simultaneously.

**Fig 6:**
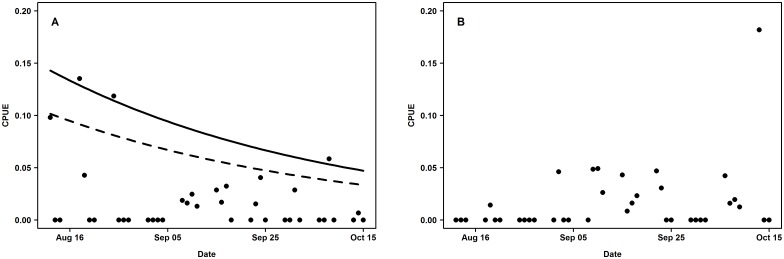
Carp and tench catch per unit effort. Catch per unit effort (CPUE: fish * rod hours ^-1^) for carp (A) and tench (B) during the entire fishing period. The solid line represents the predicted CPUE for carp if effort was constantly at the maximum daily effort during the study period (186 rod hours) and the dashed line represents the predicted CPUE for carp if effort was constantly at the median daily effort during the study period (41 rod hours).

There was a declining trend in the catch rates (assuming constant abundance) of carp over time (GLM, z_1,37_ = -1.802, p = 0.0715) (Fig 6). By contrast, there was no evidence for a corresponding trend of declining catch rates in tench over time (GLM, z_1,36_ = -0.616, p = 0.538) (Fig 6).

### Predictors of vulnerability of carp and tench

There was no support for any behavioural variable (distance swam, activity space size, time in the sublittoral, distance to the lake bottom, time at feeding sites and number of switches among feeding sites), size or energetic condition (for carp) at the time of tagging to be a predictor of speed to capture or capture probability in either carp or tench. In carp survival models (Tables 2 and 3) there were three models with an AICc below two, and all four behavioural traits (distance to the bottom, lipid percentage, time spent at feeding sites and activity space size), but not TL, were included in at least one of the best models. However, none of the behavioural variables were found to statistically explain speed of capture as the estimates of 95% confidence intervals of the hazard ratios overlapped one. In the tench survival model set (Tables 3 and 4), four models received similar support in which the distance to the lake bottom, the number of switches among feeding sites and the TL were included in at least one of the best models, while the distance swam was not. Model averaged 95% confidence intervals of the hazard ratios, however, all overlapped one, indicating no statistically significant relationship of the various traits and speed of capture in tench.

**Table 2:** The coefficients, degrees of freedom, log likelihood and Akaike metrics of the best performing survival models (ΔAICc ≤ 2) for predicting time to carp capture. The "-" symbol indicates the covariate was not included in the model.

Intercept	Distance to the lake bottom	Lipid percentage	Time spent at feeding sites	ID	Activity space size	Total length	df	Log likelihood	AICc	ΔAICc	*w*
+	-	-	-2.952	+	-	-	1	-16.878	35.9	0.00	0.188
+	-	-	-4.746	+	0.475	-	2	-15.994	36.4	0.48	0.148
+	-0.510	-0.7473	-	+	-	-	2	-16.731	37.8	1.96	0.071

**Table 3:** Model averaged parameter estimates, Standard Error (SE), hazard ratios, 95% Confidence Intervals (CI) and importance for all covariates predicting time to capture in the best survival model set (ΔAICc ≤ 2) for both carp and tench.

Species	Covariate	Estimate	SE	Hazard ratio	95% CI		Importance
*Cyprinus carpio*	Time spent at feeding sites	-3.09	2.84	0.045	1.17·10^−4^	11.92	0.83
	Activity space size	0.17	0.41	1.19	0.53	2.68	0.36
	Distance to the lake bottom	-0.09	0.29	0.92	0.52	1.61	0.17
	Lipid percentage	-0.13	0.39	0.88	0.41	1.87	0.17
*Tinca tinca*	Distance to the lake bottom	2.09	2.45	8.08	6.66·10^−2^	9.84·10^2^	0.54
	Number of switches among feeding sites	4.36	38.87	78.53	6.39·10^−32^	9.65·10^34^	0.17
	Total length	-9.20·10^−4^	7.20·10^−3^	0.99	0.996	1.013	0.15

**Table 4:** The coefficients, degrees of freedom, log likelihood and Akaike metrics of the best performing survival models (ΔAICc ≤ 2) for predicting time to tench capture. The "-" symbol indicates the covariate was not included in the model.

Intercept	Distance to the lake bottom	Distance swam	ID	Number of switches among feeding sites	Total length	df	Log likelihood	AICc	ΔAICc	*w*
+	1.773	-	+	-	-	1	-13.914	30.0	0.00	0.355
+	-	-	+	-	-	0	-15.284	30.6	0.58	0.266
+	-	-	+	0.3235	-	1	-14.728	31.6	1.63	0.157
+	1.9250	-	+	-	-0.237	2	-13.687	31.9	1.89	0.138

The best logistic regression models predicting the overall capture probability of individual carp included three models with an AICc less than two, including the null model (Tables 5 and 6). Time spent at the feeding sites, and the activity space were included in the set of models receiving the most support; however, the 95% confidence intervals of the odds ratios overlapped one, indicating no statistically significant effects. In the tench logistic regression models, there were four models that had an AICc less than two, where distance to the lake bottom, distance swam and TL were included in the best models, while the number of switches among feeding sites was not (Tables 6 and 7). Similar to the carp case, the 95% confidence intervals of the odds ratios for all covariates from the model averaged parameters overlapped one, indicating no statistically significant effect of any of the variables on the probability of capture in tench.

**Table 5:** The coefficients, degrees of freedom, log likelihood and Akaike metrics of the best performing logistic regression models (ΔAICc ≤ 2) for predicting carp capture. The "-" symbol indicates the covariate was not included in the model.

Intercept	Distance to the lake bottom	Lipid percentage	Time spent at feeding sites	Activity space size	Total length	df	Log likelihood	AICc	ΔAICc	*w*
-1.522	-	-	-1.634	-	-	2	-16.436	37.3	0.00	0.173
-1.839	-	-	-2.513	0.5792	-	3	-15.616	38.1	0.79	0.117
-1.139	-	-	-	-	-	3	-18.277	38.7	1.41	0.086

**Table 6:** Model averaged parameter estimates, Standard Error (SE), odds ratios, 95% Confidence Intervals (CI) and importance for all covariates predicting capture in the best logistic regression model set (ΔAICc ≤ 2) for both carp and tench.

Species	Covariate	Estimate	SE	Odds ratio	95% CI		Importance
*Cyprinus carpio*	Intercept	-1.53	0.72	0.22	0.05	0.93	
	Time spent at feeding sites	-1.54	1.62	0.22	8.28·10^−3^	5.60	0.77
	Activity space size	0.18	0.37	1.20	0.58	2.53	0.31
*Tinca Tinca*	Intercept	-1.04	0.56	0.35	0.11	1.13	
	Distance swam	0.59	0.75	1.81	0.40	8.13	0.55
	Distance to the lake bottom	0.63	0.82	1.88	0.36	9.73	0.54
	Total length	0.11	0.35	1.11	0.55	2.24	0.12

**Table 7:** The coefficients, degrees of freedom, log likelihood and Akaike metrics of the best performing logistic regression models (ΔAICc ≤ 2) for predicting tench capture. The "-" symbol indicates the covariate was not included in the model.

Intercept	Distance to the lake bottom	Distance swam	Number of switches among feeding sites	Total length	df	Log likelihood	AICc	ΔAICc	*w*
-0.852	-	1.231	-	-	2	-12.443	29.4	0.00	0.187
-1.205	1.315	-	-	-	2	-12.458	29.5	0.03	0.185
-1.067	0.923	0.850	-	-	3	-11.606	30.4	0.93	0.118
-1.082	-	-	-	0.865	2	-13.440	31.4	1.99	0.069

The power analysis further supported our finding that repeatable encounter-based behavioural metrics were not predictive of angling vulnerability for carp (Table 8). Either the sample size allowed for sufficient power given the estimated effect size, or the sample size needed to detect our measured effects was unrealistically high, relative to the size of any natural carp population. Concerning tench, the sample size needed to detect the significant effects given the estimated effect size for the distance swam and average distance to the lake bottom was not an unrealistic size for a natural population, suggesting the weak effects we found would become significant with a very large sample size.

**Table 8:** Sample size necessary for sufficient power (alpha = 0.05, beta = 0.8) to detect significant differences for the observed effect sizes in a logistic model predicting angling vulnerability.

Species	Behaviour	Required sample size
Carp	Time at the feeding sites	23
	Activity space size	1690
Tench	Total Length	3841
	Distance Swam	124
	Distance from the lake bottom	110

## Discussion

In disagreement with our study hypotheses, we did not detect a significant relationship between repeatable behavioural traits associated with encountering baits and angling vulnerability in two benthivorous cyprinids in an *in situ* experiment at a whole-lake scale. Similarly, we did not find support for body size (morphological trait) or energetic state (lipid content in carp) to explain angling vulnerability. These results cannot be explained by behavioural plasticity because we found the behavioural traits we used in the vulnerability models to be significantly repeatable throughout the experiment. Although some repeatability measures were low, most examined behaviours showed moderately high repeatabilities commonly reported in animal behaviour literature [81], suggesting the presence of behavioural types in both carp and tench. Though we did not find significant predictors of vulnerability in the tench, our power analysis suggests if our sample size was larger a weak effect of daily swimming distance or average distance to the lake bottom could have been detected in tench. However, the mechanism would not be encounter based as the time on fed angling sites did not predict tench vulnerability under sufficient power. Our findings instead cumulatively suggest that for benthivorous cyprinids of catchable size, encountering the bait is a necessary but insufficient condition for determining the capture probability with angling gear. It is well known that individual carp strongly differ in their angling vulnerability [1,82], with many individuals being entirely invulnerable [82] similar to the case in our experiment. This suggests behavioural traits different from those measured by the acoustic telemetry are ultimately responsible for variation in angling vulnerability in carp and tench, and we will discuss several candidates.

The key conclusion from our work depends on the study system generating data consistent with knowledge about carp and tench behavioural ecology. We found carp were more active, spent more time in the pelagic area and spent more time at the feeding sites than tench. This behaviour is consistent with carp and tench behaviour reported from semi-natural ponds where carp have been found to be more active and dominate feeding sites, outcompeting tench for food access [83–86]. Moreover, the carp in our study were larger than the tench, and it is known that with increasing body size the activity space size of fishes increases, as we observed, due to reduced predation risk [87]. We also found tench spent more time sheltering in reeds or other structures than carp, which agrees with observations of tench behaviour from ponds [88] and in the wild, particularly during daylight [89]. Reasons for the differences in carp and tench behaviours in this study are therefore likely related to species-specific differences in behavioural ecology (with carp being intrinsically more active, bold and explorative than tench), the greater domestication of carp (which tends to increase activity and boldness, [90]) and the larger body size of carp compared to the tench in our study [91,92].

Carp were initially more catchable than tench, in agreement with the less mobile and more daylight sensitive foraging patterns shown by tench [55,93]. Carp generally react strongly to groundbait presence and total catches scale proportionally to the food amount introduced by anglers [55,56], which is not the case in tench [55]. In our study, however, the initially high carp catchability quickly declined towards the low levels found in tench from the onset of the angling experiment, in line with the hypothesis of rapid hook avoidance. An alternative explanation for the decline could be temporal effects on catchability, but the shape of the decline in catch rates fully agreed with a range of previous reports of rapid hook avoidance shown by carp in experimental settings in tanks and ponds [1,82,94]. The fishing pressure we exerted on the lake was similar to reports from lake recreational fisheries for carp in Germany [69], and the fishing methods closely mirrored what cyprinid anglers for carp and tench would typically do in Europe. Overall, the species-specific behaviours shown by carp and tench, the degree of experimental fishing we exerted and the potential for rapid hook avoidance learning shown by carp are all in agreement with previous studies, suggesting our findings are unlikely methodological experimental artifacts related to the telemetry system. Moreover, the telemetry system has been carefully calibrated before and found to generate highly reliable data [46].

Consistent among-individual variation in behavioural traits is a key requirement for angling-induced selection to potentially lead to evolutionary responses in behaviours [29]. Repeatability in behaviour constitutes an upper estimate of the behaviour's heritability ([95] but see [96]). Selection on life-history traits with heritabilities in the range of 0.1–0.3 [9,11] has caused substantial evolutionary changes, and therefore the levels of repeatability that we found (0.04 to 0.41) are still high enough to potentially cause behavioural evolution in response to fishing-induced behavioural selection [97]. If, however, behaviours shown by individuals are labile, the capture process becomes random with respect to behavioural traits and no selection or evolutionary responses are possible. The very low behavioural repeatability (0.08) for the number of times carp switched among feeding sites was possibly the reason for its lack of relationship with vulnerability. By contrast, all other behaviours we measured were sufficiently and consistently variable among individuals, and consistent over very different ecological contexts. Behavioural repeatabilities in our study were not overly high, but within the average range reported in a meta-analysis of repeatabilities across animals [81]. Therefore, the lack of behavioural selection acting on telemetry-derived behaviours in the wild cannot be explained by plasticity or randomness of behavioural patterns shown by carp and tench. Following theoretical simulations [27] and empirical data generated with passive fishing gear [22,29], we expected behaviours elevating encounters with baits should elevate carp and tench angling vulnerability. However, we detected no relationship among measures that increase bait encounters, such as distance travelled, activity space size, or visits on feeding sites and angling vulnerability. We documented that the fishes rapidly started to use the feeding sites and hence were encountering the baits, but any individual variation in feeding site encounter rates nevertheless was not predictive of angling vulnerability. Consistent with the model results by Alós et al. [27] and empirical data by Olsen et al. [29] in Atlantic cod, we found no systematic selection on extent of the daily activity space ("home range"). However, the lack of relationship among swimming activity and vulnerability to capture we documented disagreed with the model results of Alós et al., [27] and the empirical findings of Alós et al., [30] in pearly razorfish fished with natural bait. In their model, Alós et al., [27] assumed the capture process is entirely encounter based and our data strongly suggest an encounter-based process is insufficient to explain angling vulnerability variation in carp and tench. In a different empirical study, Olsen and colleagues [29] reported similar findings to our own, where the vulnerability of Atlantic cod to a range of passive fishing gears was not driven by activity per se, but instead was affected by variation in diel vertical movements and the degree of shifts among shallow and deep water. These behaviours may be interpreted as related less to encounters and more to variation in risk-taking and boldness. Similarly, Philipp et al. [3] generated two largemouth bass (*Micropterus salmoides*) selection lines selected for either high or low angling vulnerability, which strongly differed in metabolism [48], parental care behaviour, aggression and catchability [38], but not swimming activity [32]. The lack of relationships among activity and vulnerability that we documented disagreed with the study hypothesis, but is consistent with most previous empirical work on the behavioural determinants of angling vulnerability in a range of species [33,34], implying species-specific or gear-specific (natural vs. artificial bait) effects. Relative to activity, other personality traits not assessed in our study such as boldness [25,28] or aggression [38] likely play a larger role in angling vulnerability than bait encounters alone.

Our focus was assessing behavioural traits associated with encounter rates for two reasons. First, encountering the bait is a necessary condition for being catchable in principle [27]. Second, the acoustic telemetry system measures encounter-based behaviours and behaviours related to space use best. By contrast, the telemetry system, with a 5 m average precision, cannot pick up fine-scale risk-taking behaviour (i.e., boldness) occurring on a given feeding site after visiting it. Previous work in ponds using two carp genotypes under controlled common-garden conditions has shown boldness affects capture vulnerability [25,82]. Klefoth et al. [25,82] measured boldness in spatially confined experimental ponds as visits on risky feeding sites spaced several metres apart, near and far from a refuge. At the population level, the domesticated genotype was bolder than wild-type carp and the domesticated fish also exhibited greater vulnerability to capture [25]. However, further individual-level analysis revealed only visits on the feeding site distant from a refuge predicted angling vulnerability; the carp showed much less variation in visits to the feeding site closer to the refuge and that variation was unpredictive of angling vulnerability (Klefoth et al. unpublished data). Notably, the two feeding spots were only several meters apart, suggesting small-scale spatial variation in risk-taking behaviour differentiates among vulnerable and invulnerable specimens in carp. We therefore suggest that boldness-related behaviours are more important traits than activity or space use for differentiating among vulnerable and invulnerable carp, and likely also tench. Therefore, future work should emphasize risk-taking related behaviours, in addition to encounters, to mechanistically understand variation in angling vulnerability in benthivorous species.

**Fig 7:**
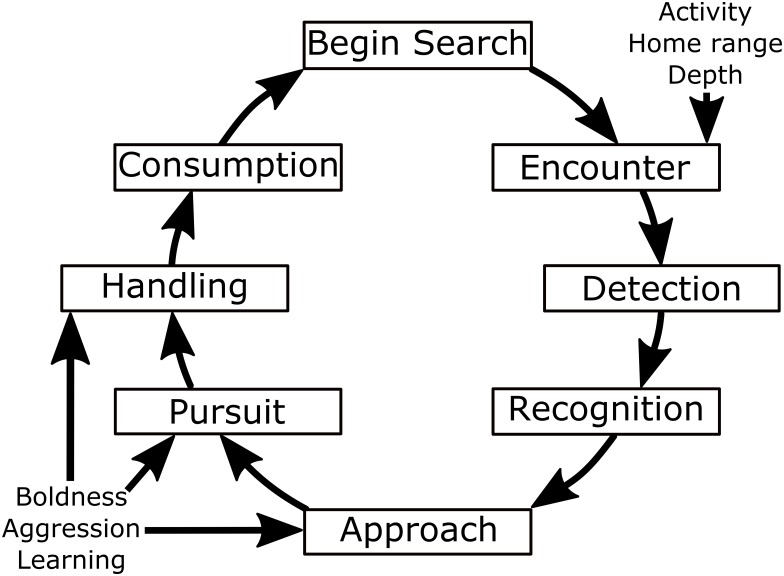
The foraging cycle. The foraging cycle adapted from Hart [103], with indications for the behaviours that are theoretically important for determining fish vulnerability to angling at specific stages of the cycle.

As encountering feeding sites was insufficient to explain the capture probability in carp and tench, it is likely behaviours further along the basic foraging cycle (Fig 7), such as those related to approach and ingestion and possibly rejection of an ingested bait, were key contributors to individual differences in angling vulnerability we documented. The same time on a feeding site can result in substantial differences in hook uptake rates when carp vary in their "trial and error" ingestion behaviour. In the laboratory, bolder, more vulnerable domesticated carp showed quicker and greater uptake rates of food items following their encounter compared to their wild-type counterparts [25]. Although all carp used in our experiment were domesticated, it is possible individuals still varied in their readiness to test, consume and expel baits on hooks [98,99]. Similar results are known from largemouth bass where highly vulnerable individuals attacked artificial lures at a higher rate than less vulnerable individuals [38]. The less vulnerable largemouth bass ingested more natural prey types, but also rejected a greater fraction of natural prey after ingestion [100]. Accordingly, individual carp may also vary in differentiating hooked baits from unhooked ones. Indeed, upon hook recognition, carp are known to spit the hook without becoming captured (see videos in association with [82]), and the general ability to recognize hooks through experiential or observational learning [1,25] could differentiate vulnerable from invulnerable individuals. Carp skilled in hook-avoidance may also be risk averse as risk-taking behaviour was negatively related to cognitive ability in carp via coping styles [101]. It is also possible that individual carp differ in their mobility after sucking in the bait, with only the ones showing the greater mobility swimming against the fixed lead, yielding a hooking event. Finally, carp of the same size are known to vary in competitive scrambling ability [102], which might also have contributed to variation in vulnerability in our study without being related to encounter based behaviours.

Morphological and physiological state traits such as size in the carp and the tench or lipid content in the carp did not predict angling vulnerability. Size selectivity is a common feature in recreational fisheries where larger individuals are typically preferentially captured compared to smaller ones [51]. We found no size-selection was present in either species, but our sample contained rather large bodied individuals. Also, with respect to gape limitations, our fishing tackle was tuned to specifically to target the full size range of tagged carp and tench. After having excluded gear selectivity effects in our work, we only would expect size to be related to vulnerability if size was correlated to another (most likely behavioural) trait linked to angling vulnerability that was unmeasured by the telemetry array. However, size was not correlated to any measured behaviours, and given the lack of selection operating on size in general it is unlikely that size (within the size ranged tested in this study) was related to any key behavioural trait (e.g., boldness, hook avoidance) predicting vulnerability in carp or tench [25].

The reasons for the lack of relationship between lipid content and vulnerability in the carp are less clear. The lipid content we measured is predictive of the whole body energy density in carp [61]. We expected that a fish with low energy density is required to forage more, implying increased encounters with baits or greater uptake of free baits once encountered. Both corn and boilies are nutrient rich, well digestible and facilitate growth [57,67]. Three non-mutually exclusive reasons might explain the lack of relationship between lipid content and angling vulnerability. First, fish varying in lipid content may have been similarly able to rapidly develop hook avoidance behaviour [82]. Second, the lipid content may have been sufficient in all individuals to negate differences in condition. In our sample the lowest energy density corresponded to 5.3 MJ *Kg^-1^ [61], which was above the threshold (4 kJ g^-1^) considered poor condition [104]. Third, the time from lipid content assessment at tagging to the assessment of vulnerability to angling included several weeks or months, in which the fish might have altered the lipid content. The most likely case is that the fish were simply all in a sufficient energetic state, rendering the lipid content measure unpredictive of angling vulnerability.

In conclusion, the behavioural mechanisms driving angling vulnerability in freshwater benthivorous fish seem unrelated to encounter rates alone. Encounters with baits are thus a necessary, but insufficient condition for affecting vulnerability to angling. In carp and tench, we would predict no fisheries-induced selection on activity or space use behaviours to happen. Candidate traits on which behavioural selection might act include boldness and aggression as well as learning ability in the context of identifying baited hooks. Future investigations into individual differences in boldness, cognition and coping styles and the ability to distinguish and avoid fishing baits may therefore prove productive for understanding the mechanisms differentiating vulnerable and invulnerable individuals. Such investigations require additional tools to observe behaviour in the wild at finer scales such as underwater cameras, simultaneously with reality mining tools such as high-resolution acoustic telemetry or PIT tag arrays. A possible alternative is using an experimental approach in naturalized ponds where behaviour on feeding spots could more readily be quantified at finer spatial scales. Our work thus does not suggest behaviour is unimportant in the capture process in angling, but rather the behavioural traits determining vulnerability must be found outside basal encounter based mechanisms in the domains of boldness, aggression, cognition and competitive ability.

## Supporting information

S1 Appendix An assessment of lake conditions, description of angling methods, description of behavioural calculations, a summary of individual fish used in vulnerability models, a partitioning of among- and within-individual variances for each behaviour, example positions for live fish and for lost tags and a submerged macrophyte map of Kleiner Döllnsee. (DOCX)

S2 Appendix Trace plots of within- and between-individual covariances estimations for six carp behaviours, each partitioned across days using univariate mixing models fit with Markov Chain Monte Carlo procedures. (PDF)

S3 Appendix Trace plots of within- and between-individual covariances estimations for six tench behaviours, each partitioned across days using univariate mixing models fit with Markov Chain Monte Carlo procedures. (PDF)

S1 Video A video of five days of carp movement in Kleiner Döllnsee during the angling phase of the experiment, distinguishing captured and uncaptured individuals. (MP4)

S2 Video A video of five days of tench movement in Kleiner Döllnsee during the angling phase of the experiment, distinguishing captured and uncaptured individuals. (M4V)
