# Supplementary material for: Encountering a bait is necessary but insufficient to explain individual variability in vulnerability to angling in two freshwater benthivorous fish in the wild
Source: PLoS One. 2017 Mar 16;12(3):e0173989. doi: 10.1371/journal.pone.0173989 (PMC5354434; doi:10.1371/journal.pone.0173989)
Supplement: S1 Appendix — (DOCX) [file pone.0173989.s001.docx]

**Supplementary Material**

**Assessment of lake conditions**

To create bathymetric and submerged macrophyte coverage maps and relate fish positions to habitat conditions in the lake, echograms were recorded on July 16, 2014 with a split beam EY60 echosounder (Simrad, Norway) connected to an ES 120-7C circular composite transducer fixed 0.4 m below the water surface. The system was operated on 120 kHz. Transects were made along the north south axis of the lake spaced 50 m apart and the positions of the measurements were recorded simultaneously every second with a handheld GeoXH dGPS receiver (Trimble, California, USA) with metre precision.

The echograms were processed with Sonar 5 Pro software (1). The lake bottom was selected from the echogram using the Macrophyte analysis tool. Macrophyte base detection with the best-candidate approach was used to find the lake bottom (parameters: pre-filter height = 1, pre-filter width = 15, threshold = -32 dB, margin = 0 m, minimum range = 0.1 m, maximum range = 10 m, start level = -32 dB, stop level = -43 dB, minimum length = 2, minimum gate = 5 m, post-filter height = 1, post-filter width = 5 and minimum target = 5 m). To find the macrophyte tops, the top detection tool with the best-candidate approach was used and the parameters from the base detection were applied. Errors in the lake bottom and macrophyte tops were manually corrected and the depths with their coordinates were further analysed in R version 3.2.1 (CRAN).

As there were multiple pings in the echogram each second, first depth and macrophyte coverage were averaged for each gps coordinate pair in the transect. To create the bathymetric map a variogram was fit to the depth measurements along the transect, which was then used for interpolation on a metre by metre grid of the lake by ordinary kriging considering a maximum distance of 100 m (package gstat version 1.0.26; (2)). The submerged macrophyte map was also created with ordinary kriging applied in the same way.

**Angling methods**

Experimental angling occurred using standard carp fishing gear with a bolt rig supposed to facilitate self hooking (3). We used two to three rods (BeastMaster BX 12 2 3/8 16 T/C, Shimano, Osaka, Japan) per angler, each with a BaitRunner X-Aero 8000AR reel (Shimano, Osaka, Japan). The leader length and hair length were standardized to 16 cm and 3 cm from the eye of the hook respectively. We used the size 6 G-carp superhook (Gamakatsu, Tacoma, USA) and an 85 g fixed lead weight. As bait, boiled feed-corn (2-3 pieces) was used on one rod and 14 mm diameter boilies (4) (M&M Baits Neuenkirchen-Vörden, Germany) (fishmeal or birdseed mix; 1-2 pieces) were used on the other and the baits were reversed between rods each day standardizing bait location. Boilies are typical baits used by carp anglers and are made from boiled and hardened mixtures of meal types and eggs (see (4) for details). Baits were cast to land at the feeding site being angled. During the first three weeks of fishing, all eight feeding sites from the feeding experiment (Fig 2) were baited with 2 kg of boiled feed corn and 1 kg of pelleted boilies in the morning and in the evening. During the remaining weeks of fishing, the feeding was reduced to only the four sites that were fished, and the food was generally spread by hand as would be typical in angling, rather than introduced in a bag (which was initially used to verify the food was eaten by the fish, which was always the case). The food amount was reduced because of concern that excess food was satiating the fish and limiting catch rates. Sites were still fed regardless of whether an angler was present to maintain attractiveness of that site.

If a carp or tench was captured during the daytime, it was sampled and released immediately. If the carp or tench was captured in the night it was held in a so-called carp sack, as is typical in carp angling (5), until the morning when it was enumerated and released. The behaviour of bagged carp returns to normal within 12 hours (5).

**Calculation of fish behaviours**

Distance swam was calculated as the sum of the Euclidean distances in metres between consecutive positions. Large gaps in detection times create substantial uncertainty about the distance travelled and therefore differences in detection rates can bias the estimation of swimming distance. For comparable distance swam measurements among individuals, we did not add distances between consecutive positions spanning more than 335 seconds, which is a time period in which there is a very high detection probability. The 335 second time period is calibrated from detection rates of beacon transmitters fixed underneath the hydrophones in the lake. Additionally in our distance calculation, distances below 5 m (the error rate of the telemetry system (6)) were eliminated from the calculation to reduce the accumulation of measurement error when fish are not moving. The remaining distances were summed daily for each individual.

The pressure sensor in the acoustic tags was used to measure the vertical position of the fish in the water column and subsequently the daily average distance from the bottom of the lake. The acoustic transmitters were programmed to transmit temperature information instead of depth once per minute and accordingly positions without depth information were eliminated from the calculation. For each remaining positions, the depth of the fish was subtracted from the corresponding lake depth and then averaged daily for each individual.

The time in the sublittoral was calculated as the number of seconds that the fish was in a location without submerged macrophytes or reeds as inferred from the habitat maps that we estimated (Fig S.5). As before, there was some uncertainty about when a fish had truly left the sublittoral area to move back to the refuge. Therefore, if a fish was in the sublittoral area and then not detected for more than 335 seconds (for determination of this interval see above), it was considered likely to have left the sublittoral area by the end of that period, and 335 seconds was added to the daily time spent in the sublittoral zone for that individual fish. The total time in the pelagic was summed daily for each individual. Times between two consecutive positions recorded from one day to the next were cut at midnight and the two durations were added to the appropriate days.

To estimate the activity space size during any given day, fixed normal kernel densities were calculated for each individual. When fewer than 30 positions were recorded for an individual in a day the activity space was not estimated because of the small sample size (7). Our kernel estimation was based on a 200 by 104 cell grid, with a cell size of 5.78 m and estimated in R using the *adehabitatHR* package (8). The smoothing parameter *h* was set to 10 m. We then calculated the area in m^2^ of the 50% utilization distribution by taking the 50% volume contour as our measurement of activity space. We also calculated the home range of the population of carp collectively during the exploration, lake manipulation and angling phases to see how the feeding sites affected the distribution of the carp in the lake overall. We did the same for the entire population of tench as well, excluding the exploration phase as no tench were acoustically tagged at that time.

A fish was considered to be within the GPS-positioned feeding site, while it was within three times the precision of the telemetry system (15 m) (6). The duration of a feeding site visit was calculated starting from the initial detection at the feeding site until the first detection outside of the 15 m radius. If the time between the last detection within the 15 m radius and the first detection outside of the radius was greater than 335 seconds, only 335 seconds were added to the duration of the feeding site visit. Feeding site visit times were then summed daily for each individual. Times between two consecutive positions recorded from one day to the next were again cut at midnight and the two durations were added to the appropriate days.

Finally, a tally was made each time a feeding site was visited that was different from the feeding site of the previous visit. These tallies were summed daily for each individual to calculate the number of switches among the feeding sites.

**References**

1. Balk H, Lindem T. Sonar4 and Sonar5-Pro Post Processising Systems Operator Manual Version 5.9.7. Oslo, Norway: University of Oslo; 2007. p. 435p.

2. Pebesma EJ. Multivariable geostatistics in S: The gstat package. Comput Geosci. 2004;30(7):683–91.

3. Rapp T, Cooke SJ, Arlinghaus R. Exploitation of specialised fisheries resources: The importance of hook size in recreational angling for large common carp (*Cyprinus carpio* L.). Fish Res. 2008;94(1):79–83.

4. Niesar M, Arlinghaus R, Rennert B, Mehner T. Coupling insights from a carp, *Cyprinus carpio*, angler survey with feeding experiments to evaluate composition, quality and phosphorus input of groundbait in coarse fishing. Fish Manag Ecol. 2004;11(3–4):225–35.

5. Rapp T, Hallermann J, Cooke SJ, Hetz SK, Wuertz S, Arlinghaus R. Physiological and behavioural consequences of capture and retention in carp sacks on common carp (Cyprinus carpio L.), with implications for catch-and-release recreational fishing. Fish Res. 2012;125–126:57–68.

6. Baktoft H, Zajicek P, Klefoth T, Svendsen JC, Jacobsen L. Performance assessment of two whole-lake acoustic positional telemetry systems - is reality mining of free-ranging aquatic animals technologically possible? PLoS One. 2015;1–20.

7. Seaman DE, Millspaugh JJ, Kernohan BJ, Brundige GC, Raedeke KJ, Gitzen RA. Effects of sample size on kernel home range estimates. J Wildl Manage. 1999;63(2):739–47.

8. Calenge C. Home Range Estimation in R : the adehabitatHR Package. R vignette. 2011;1–60.

**Table S.1.** Telemetry data recorded for all carp included in the final analysis testing the relationship between behaviour and vulnerability to angling.

| ID | Transmitter Model | Bust Rate (s) | Total Length (mm) | Wet Mass (g) | Day Released | Days tracked | Daily Detections | | | | |
| --- | --- | --- | --- | --- | --- | --- | --- | --- | --- | --- | --- |
|  |  |  |  |  |  |  | Mean ± sd | Minimum | Maximum | Mean % | Maximum % |
| 70600 | MM-M-TP-16-50 | 5 | 509 | 2450 | 2015.06.12 | 123 | 734.2 ± 925 | 0 | 4389 | 4.2 | 25.4 |
| 68200 | MM-M-TP-16-50 | 5 | 522 | 2439 | 2015.06.12 | 126 | 657.6 ± 868 | 0 | 3136 | 3.8 | 18.1 |
| 62400 | MM-M-TP-16-50 | 5 | 722 | 6934 | 2015.06.13 | 108 | 603.1 ± 670 | 0 | 3092 | 3.5 | 17.9 |
| 61600 | MM-M-TP-16-50 | 5 | 573 | 3323 | 2015.06.13 | 125 | 727.3 ± 761 | 0 | 2999 | 4.2 | 17.4 |
| 60700 | MM-M-TP-16-50 | 5 | 519 | 2731 | 2015.06.13 | 15 | 1378.4 ± 1511 | 0 | 6365 | 8.0 | 36.8 |
| 63700 | MM-M-TP-16-50 | 5 | 492 | 1783 | 2015.06.13 | 125 | 1490 ±1721 | 0 | 6037 | 8.6 | 34.9 |
| 63600 | MM-M-TP-16-50 | 5 | 430 | 1171 | 2015.09.05 | 40 | 1287.1 ±926 | 0 | 3215 | 7.4 | 18.6 |
| 65800 | MM-M-TP-16-50 | 5 | 707 | 5872 | 2015.09.05 | 40 | 954.5 ± 783 | 1 | 2663 | 5.5 | 15.4 |
| 60200 | MM-M-TP-16-50 | 5 | 615 | 2596 | 2015.06.13 | 108 | 384.9 ± 648 | 0 | 3571 | 2.2 | 20.7 |
| 60600 | MM-M-TP-16-50 | 5 | 608 | 3310 | 2015.06.12 | 122 | 1118.7 ± 1073 | 0 | 4663 | 6.5 | 27.0 |
| 61000 | MM-M-TP-16-50 | 5 | 555 | 3170 | 2015.06.12 | 126 | 1325.1 ± 1231 | 0 | 4302 | 7.7 | 24.9 |
| 61100 | MM-M-TP-16-50 | 5 | 643 | 4127 | 2015.06.13 | 125 | 789.1 ± 824 | 0 | 3116 | 4.6 | 18.0 |
| 61400 | MM-M-TP-16-50 | 5 | 628 | 4028 | 2015.06.13 | 125 | 1374 ± 1287 | 0 | 4623 | 8.0 | 26.8 |
| 62100 | MM-M-TP-16-50 | 5 | 644 | 4779 | 2015.06.13 | 125 | 347.6 ± 487 | 0 | 2395 | 2.0 | 13.9 |
| 62500 | MM-M-TP-16-50 | 5 | 653 | 4429 | 2015.06.13 | 125 | 604.5 ± 894 | 0 | 4632 | 3.5 | 26.8 |
| 64900 | MM-M-TP-16-50 | 5 | 623 | 4083 | 2015.06.13 | 125 | 365.3 ± 535 | 0 | 2996 | 2.1 | 17.3 |
| 65500 | MM-M-TP-16-50 | 5 | 588 | 3367 | 2015.06.13 | 125 | 265.3 ± 446 | 0 | 2279 | 1.5 | 13.2 |
| 67700 | MM-M-TP-16-50 | 5 | 473 | 2451 | 2015.06.12 | 126 | 1167.5 ± 1505 | 0 | 7754 | 6.8 | 44.9 |
| 67800 | MM-M-TP-16-50 | 5 | 563 | 2915 | 2015.06.12 | 126 | 392.4 ± 572 | 0 | 2529 | 2.3 | 14.6 |
| 69400 | MM-M-TP-16-50 | 5 | 434 | 1354 | 2015.06.12 | 126 | 612 ± 780 | 0 | 3399 | 3.5 | 19.7 |
| 69500 | MM-M-TP-16-50 | 5 | 540 | 3227 | 2015.06.12 | 126 | 1516.2 ± 1806 | 0 | 6179 | 8.8 | 35.8 |
| 69600 | MM-M-TP-16-50 | 5 | 458 | 1647 | 2015.06.12 | 126 | 1352.5 ±1530 | 0 | 5762 | 7.8 | 33.3 |
| 69800 | MM-M-TP-16-50 | 5 | 519 | 2041 | 2015.06.12 | 126 | 749.6 ± 897 | 0 | 3790 | 4.3 | 21.9 |
| 70200 | MM-M-TP-16-50 | 5 | 508 | 2213 | 2015.06.12 | 126 | 492.8 ± 732 | 0 | 3323 | 2.9 | 19.2 |
| 62300 | MM-M-TP-16-50 | 5 | 486 | 1840 | 2015.09.05 | 40 | 1537.5 ± 1114 | 0 | 4224 | 8.9 | 24.4 |
| 63200 | MM-M-TP-16-50 | 5 | 455 | 1850 | 2015.09.05 | 40 | 768.2 ± 616 | 0 | 1964 | 4.4 | 11.4 |
| 63500 | MM-M-TP-16-50 | 5 | 474 | 1530 | 2015.09.05 | 40 | 310.8 ± 416 | 0 | 1555 | 1.8 | 9.0 |
| 63900 | MM-M-TP-16-50 | 5 | 505 | 2130 | 2015.09.05 | 40 | 686.1 ± 702 | 0 | 2869 | 4.0 | 16.6 |
| 64800 | MM-M-TP-16-50 | 5 | 510 | 1760 | 2015.09.05 | 40 | 1955 ± 1461 | 0 | 5954 | 11.3 | 34.5 |
| 65000 | MM-M-TP-16-50 | 5 | 470 | 1700 | 2015.09.05 | 40 | 256.2 ± 380 | 0 | 1384 | 1.5 | 8.0 |
| 67200 | MM-M-TP-16-50 | 5 | 550 | 2633 | 2015.09.05 | 40 | 1173.6 ± 765 | 0 | 2714 | 6.8 | 15.7 |
| 70400 | MM-M-TP-16-50 | 5 | 550 | 2810 | 2015.09.05 | 40 | 1142.2 ±940 | 0 | 3050 | 6.6 | 17.7 |
| 74000 | MM-M-TP-16-50 | 5 | 437 | 1250 | 2015.09.05 | 40 | 1337 ± 987 | 0 | 3130 | 7.7 | 18.1 |

**Table S.2.** Telemetry data recorded for all tench included in the final analysis testing the relationship between behaviour and vulnerability to angling. The source KD is Kleiner Döllnsee, and the source GV is Großer Vätersee.

| ID | Transmitter Model | Burst Rate (s) | Total Length (mm) | Wet Mass (g) | Source | Release Date | Days of Tracking | Daily Detections | | | | |
| --- | --- | --- | --- | --- | --- | --- | --- | --- | --- | --- | --- | --- |
|  |  |  |  |  |  |  |  | Mean ± sd | Minimum | Maximum | Mean % | Maximum % |
| 74600 | MM-M-TP-11-28 | 35 | 434 | 1185 | Oder | 2015.08.14 | 62 | 195.6 ± 179 | 0 | 702 | 7.9 | 28.4 |
| 77700 | MM-M-TP-11-28 | 35 | 430 | 1240 | Oder | 2015.08.14 | 62 | 319.1 ± 195 | 6 | 917 | 12.9 | 37.2 |
| 75700 | MM-M-TP-11-28 | 35 | 501 | 1763 | KD | 2015.08.19 | 57 | 387.6 ± 284 | 0 | 999 | 15.7 | 40.5 |
| 76900 | MM-M-TP-11-28 | 35 | 508 | 1950 | Oder | 2015.08.26 | 50 | 100.4 ± 107 | 0 | 396 | 4.1 | 16.0 |
| 75100 | MM-M-TP-11-28 | 35 | 477 | 1949 | Oder | 2015.08.14 | 62 | 262.3 ± 287 | 0 | 1056 | 10.6 | 42.8 |
| 77700 | MM-M-TP-11-28 | 35 | 430 | 1240 | Oder | 2015.08.14 | 62 | 319.1 ± 195 | 6 | 917 | 12.9 | 37.2 |
| 77400 | MM-M-TP-11-28 | 35 | 523 | 1777 | KD | 2015.09.03 | 42 | 382.8 ± 240 | 0 | 920 | 15.5 | 37.3 |
| 75700 | MM-M-TP-11-28 | 35 | 501 | 1763 | KD | 2015.08.19 | 57 | 387.6 ± 284 | 0 | 999 | 15.7 | 40.5 |
| 75000 | MM-M-TP-11-28 | 35 | 455 | 1506 | Oder | 2015.08.14 | 62 | 119.8 ± 128 | 0 | 744 | 4.9 | 30.1 |
| 74400 | MM-M-TP-11-28 | 35 | 408 | 1028 | Oder | 2015.08.14 | 62 | 52.7 ± 79 | 0 | 397 | 2.1 | 16.1 |
| 74500 | MM-M-TP-11-28 | 35 | 476 | 2099 | Oder | 2015.08.14 | 62 | 224.1 ± 198 | 0 | 773 | 9.1 | 31.3 |
| 74700 | MM-M-TP-11-28 | 35 | 501 | 1850 | Oder | 2015.08.14 | 62 | 218.5 ± 178 | 0 | 743 | 8.9 | 30.1 |
| 75200 | MM-M-TP-11-28 | 35 | 404 | 1024 | Oder | 2015.08.14 | 62 | 57.8 ± 79 | 0 | 323 | 2.3 | 13.1 |
| 75300 | MM-M-TP-11-28 | 35 | 478 | 1783 | Oder | 2015.08.14 | 62 | 140.5 ± 149 | 0 | 667 | 5.7 | 27.0 |
| 75500 | MM-M-TP-11-28 | 35 | 422 | 1177 | Oder | 2015.08.26 | 50 | 146.8 ±200 | 0 | 999 | 5.9 | 40.5 |
| 75600 | MM-M-TP-11-28 | 35 | 465 | 1399 | KD | 2015.08.19 | 57 | 197.8 ± 191 | 0 | 745 | 8.0 | 30.2 |
| 75800 | MM-M-TP-11-28 | 35 | 450 | 1309 | Oder | 2015.08.26 | 50 | 87.6 ± 79 | 0 | 296 | 3.5 | 12.0 |
| 75900 | MM-M-TP-11-28 | 35 | 476 | 1585 | Oder | 2015.08.26 | 50 | 127.9± 327 | 0 | 1413 | 5.2 | 57.3 |
| 76000 | MM-M-TP-11-28 | 35 | 380 | 846 | Oder | 2015.08.26 | 50 | 32.9 ± 67 | 0 | 297 | 1.3 | 12.0 |
| 76100 | MM-M-TP-11-28 | 35 | 467 | 1552 | Oder | 2015.08.26 | 50 | 197.5 ± 151 | 0 | 620 | 8.0 | 25.1 |
| 76300 | MM-M-TP-11-28 | 35 | 498 | 1863 | Oder | 2015.08.26 | 50 | 425.7 ± 287 | 0 | 1048 | 17.2 | 42.5 |
| 76400 | MM-M-TP-11-28 | 35 | 451 | 1349 | Oder | 2015.08.26 | 50 | 178.7 ± 208 | 0 | 892 | 7.2 | 36.1 |
| 76500 | MM-M-TP-11-28 | 35 | 481 | 1954 | Oder | 2015.08.26 | 50 | 198.9 ± 211 | 0 | 819 | 8.1 | 33.2 |
| 76600 | MM-M-TP-11-28 | 35 | 466 | 1258 | Oder | 2015.08.26 | 50 | 129.7 ± 192 | 0 | 813 | 5.3 | 32.9 |
| 76700 | MM-M-TP-11-28 | 35 | 446 | 1456 | Oder | 2015.08.26 | 50 | 78.8 ± 78 | 0 | 353 | 3.2 | 14.3 |
| 77600 | MM-M-TP-11-28 | 35 | 371 | 736 | GV | 2015.07.17 | 90 | 32 ± 75 | 0 | 436 | 1.3 | 17.7 |
| 77900 | MM-M-TP-11-28 | 35 | 433 | 1318 | GV | 2015.07.17 | 90 | 115.5 ± 135 | 0 | 709 | 4.7 | 28.7 |

**Tabl****e S.3.** The variance partitioning for carp behaviours used in the calculation of repeatability.

| Behavioural Trait | Among Individual Variance | | | Within Individual Variance | | | Repeatability | | |
| --- | --- | --- | --- | --- | --- | --- | --- | --- | --- |
|  | Estimate | Lower 95% CI | Upper 95% CI | Estimate | Lower 95% CI | Upper 95% CI | Estimate | Lower 95% CI | Upper 95% CI |
| Distance from the Lake Bottom | 0.44 | 0.28 | 0.64 | 0.95 | 0.90 | 0.99 | 0.31 | 0.24 | 0.41 |
| Distance Swam | 7.98 | 4.96 | 11.82 | 25.51 | 24.08 | 26.97 | 0.23 | 0.16 | 0.32 |
| Time within 15 m of feeding sites | 13.51 | 6.17 | 22.75 | 73.33 | 66.64 | 79.75 | 0.14 | 0.08 | 0.23 |
| Activity space size | 9.34 | 5.45 | 13.54 | 25.24 | 23.77 | 26.72 | 0.26 | 0.18 | 0.35 |
| Number of switches among feeding sites | 0.72 | 0.32 | 1.31 | 6.51 | 5.86 | 7.09 | 0.08 | 0.04 | 0.14 |
| Time in the sublittoral zone | 8.06 | 4.80 | 11.40 | 26.99 | 25.51 | 28.40 | 0.23 | 0.16 | 0.30 |

**Table S.4.** The variance partitioning for tench behaviours used in the calculation of repeatability

| Behavioural Trait | Among Individual Variance | | | Within Individual Variance | | | Repeatability | | |
| --- | --- | --- | --- | --- | --- | --- | --- | --- | --- |
|  | Estimate | Lower 95% CI | Upper 95% CI | Estimate | Lower 95% CI | Upper 95% CI | Estimate | Lower 95% CI | Upper 95% CI |
| Distance from the Lake Bottom | 0.30 | 0.15 | 0.47 | 0.81 | 0.75 | 0.87 | 0.26 | 0.16 | 0.37 |
| Distance Swam | 11.99 | 5.77 | 19.28 | 48.99 | 44.38 | 53.64 | 0.19 | 0.11 | 0.28 |
| Time within 15 m of feeding sites | 49.29 | 21.92 | 79.71 | 114.82 | 97.39 | 133.33 | 0.27 | 0.16 | 0.39 |
| Activity space size | 18.82 | 8.74 | 31.74 | 51.15 | 46.30 | 56.65 | 0.25 | 0.15 | 0.38 |
| Number of switches among feeding sites | 3.46 | 1.60 | 5.63 | 5.93 | 4.93 | 6.90 | 0.27 | 0.17 | 0.40 |
| Time in the sublittoral zone | 15.78 | 7.69 | 25.27 | 63.05 | 57.52 | 68.71 | 0.19 | 0.11 | 0.29 |

**
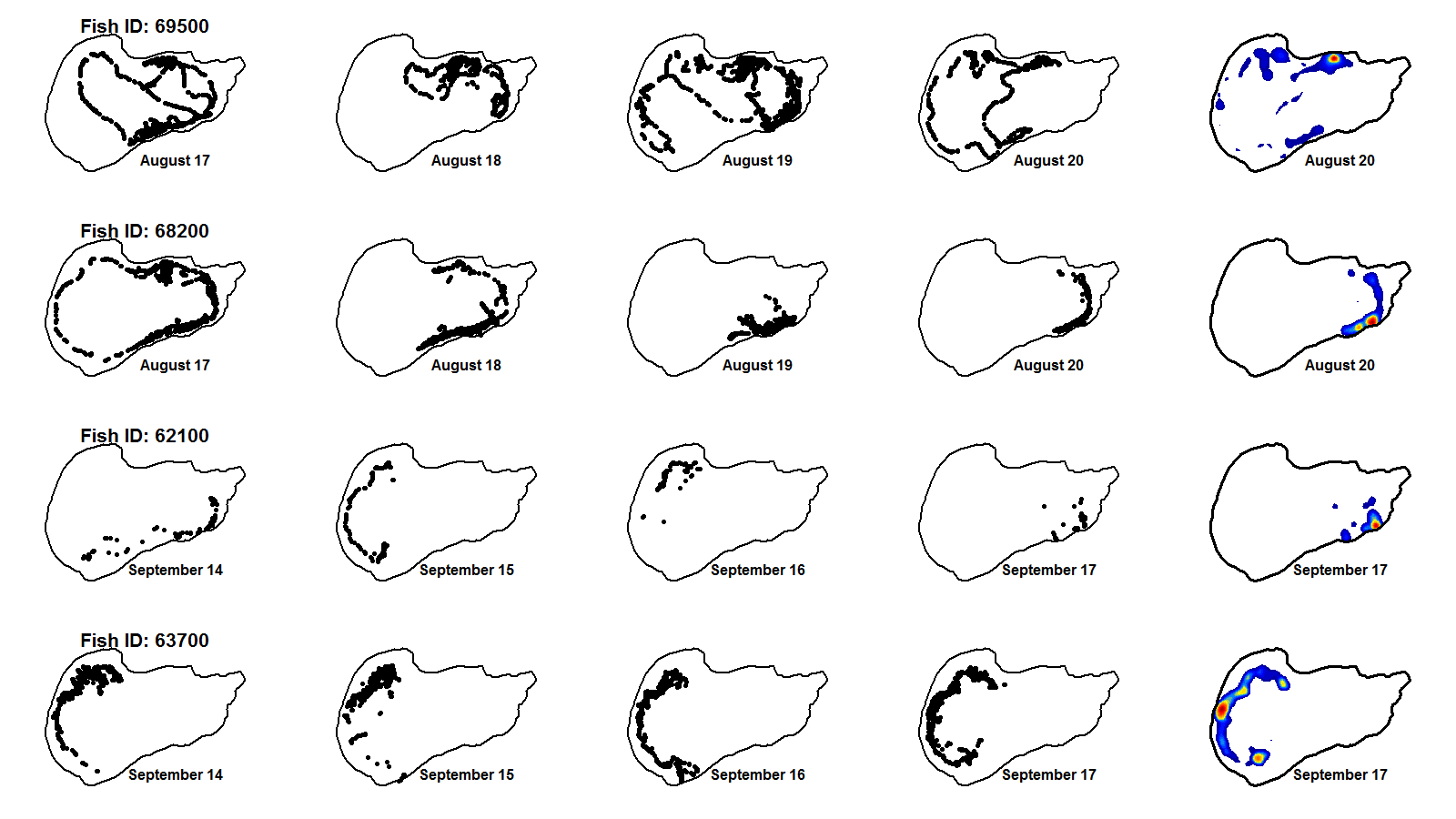
**

**Figure S.1.** Examples of the data for four individual carp over four days. The kernel utilization density calculated for one day is shown in the last panel on the right for each fish.

**
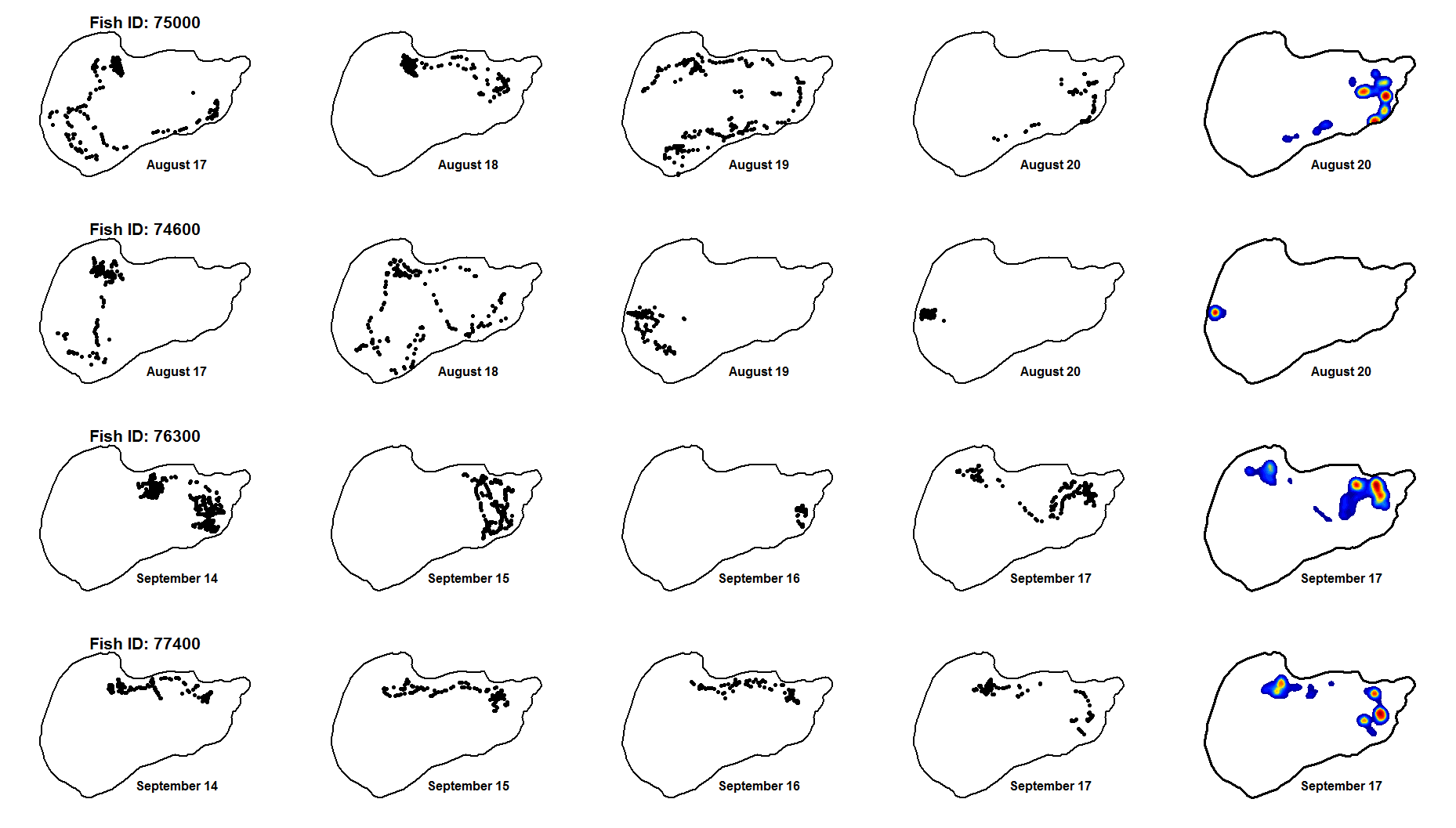
**

**Figure S.2.** Examples of the data for four individual tench over four days. The kernel utilization density calculated for one day is shown in the last panel on the right for each fish.


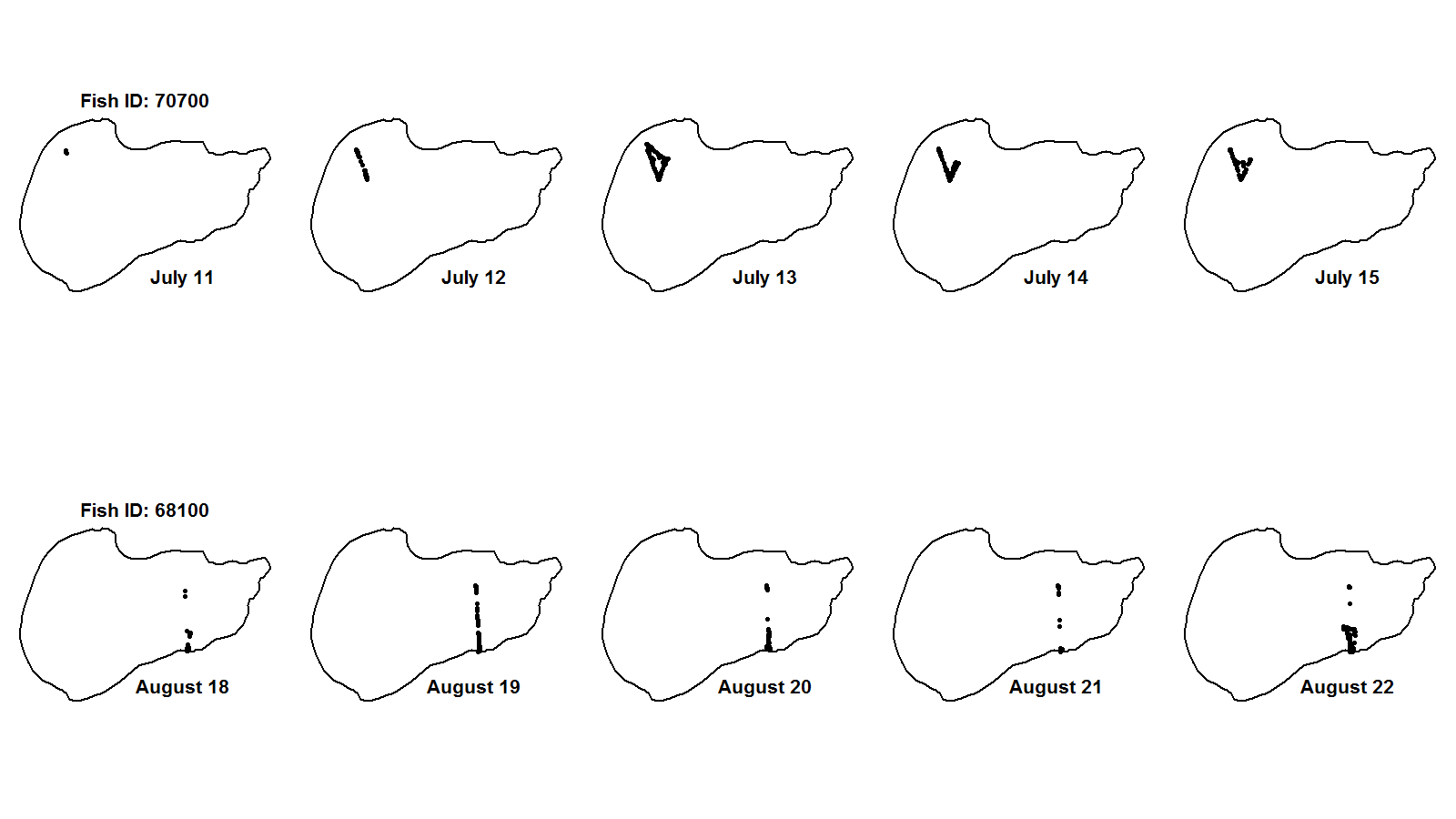


**Figure S.3.** Examples of position calculated from a tag sitting on the bottom of the lake as a result of either tag loss or fish death. This situation can be identified visually by the consistency of positions across days and the geometric patterns of the positions as a result of systematic error in positioning.

**
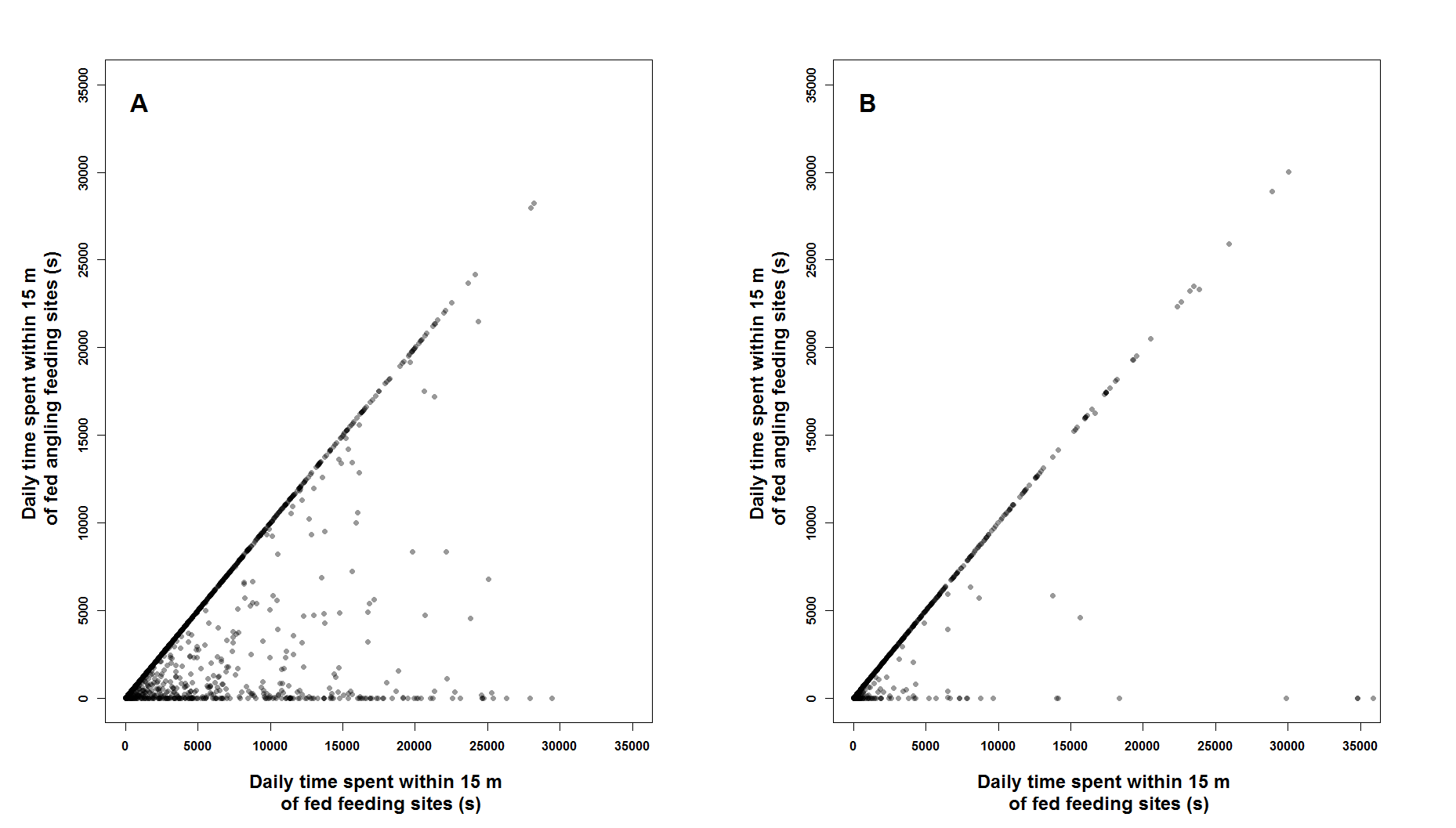
**

**Figure S.4.** The relationship between the daily time spent at the fed feeding spots and the daily time spent at the angling sites while they were being fed for carp (A) and tench (B) The time period covers July 3 to October 15 (i.e the feeding manipulation and angling phases).

**
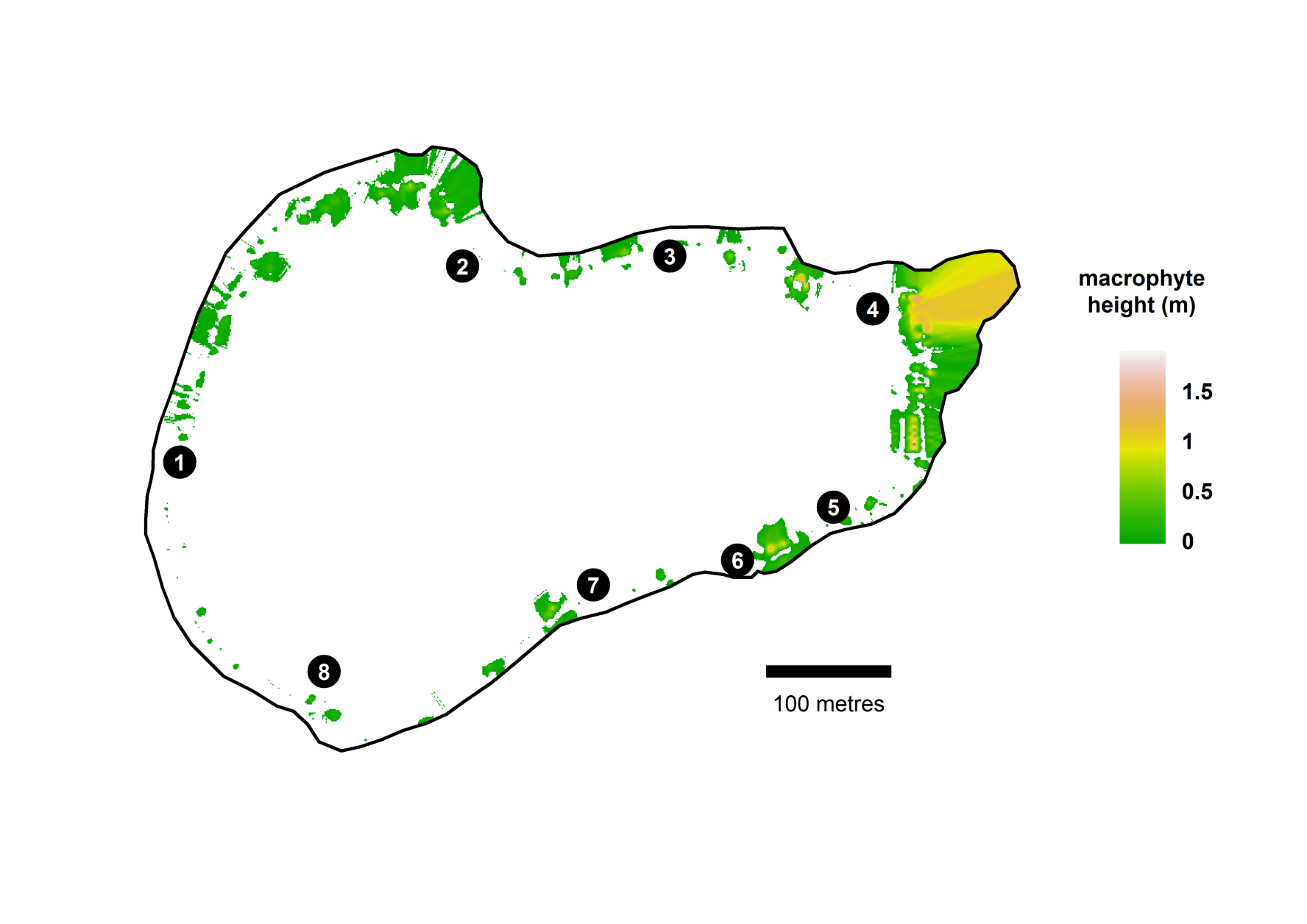
**

**Figure S.5.** The submerged macrophyte height in Kleiner Döllnsee estimated from a bathymetric survey and the location of eight feeding sites around the lake.
