# Supplementary material for: Encountering a bait is necessary but insufficient to explain individual variability in vulnerability to angling in two freshwater benthivorous fish in the wild
Source: PLoS One. 2017 Mar 16;12(3):e0173989. doi: 10.1371/journal.pone.0173989 (PMC5354434; doi:10.1371/journal.pone.0173989)
Supplement: S2 Appendix — (PDF) [file pone.0173989.s002.pdf]

Distance to the lake bottom

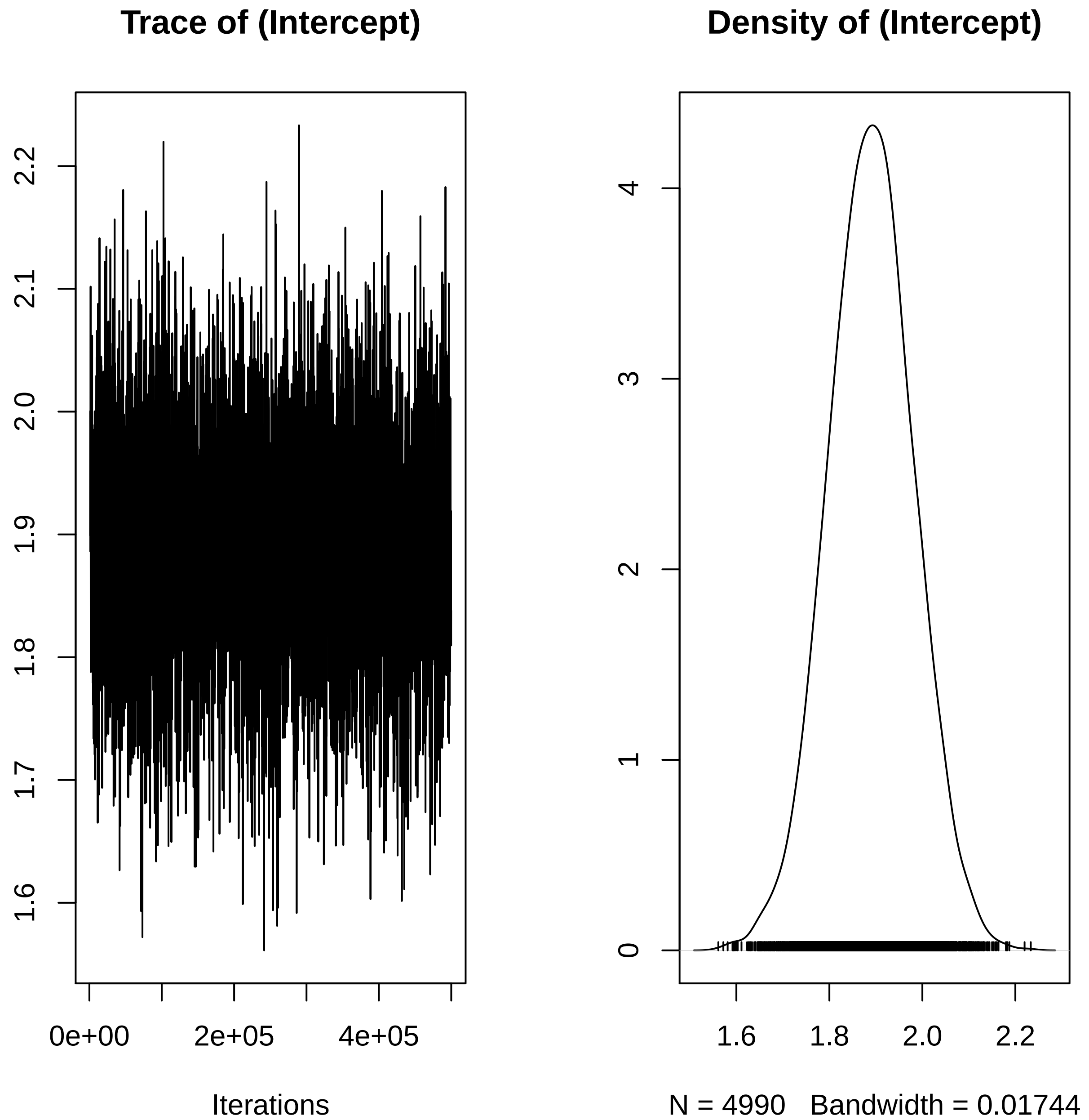

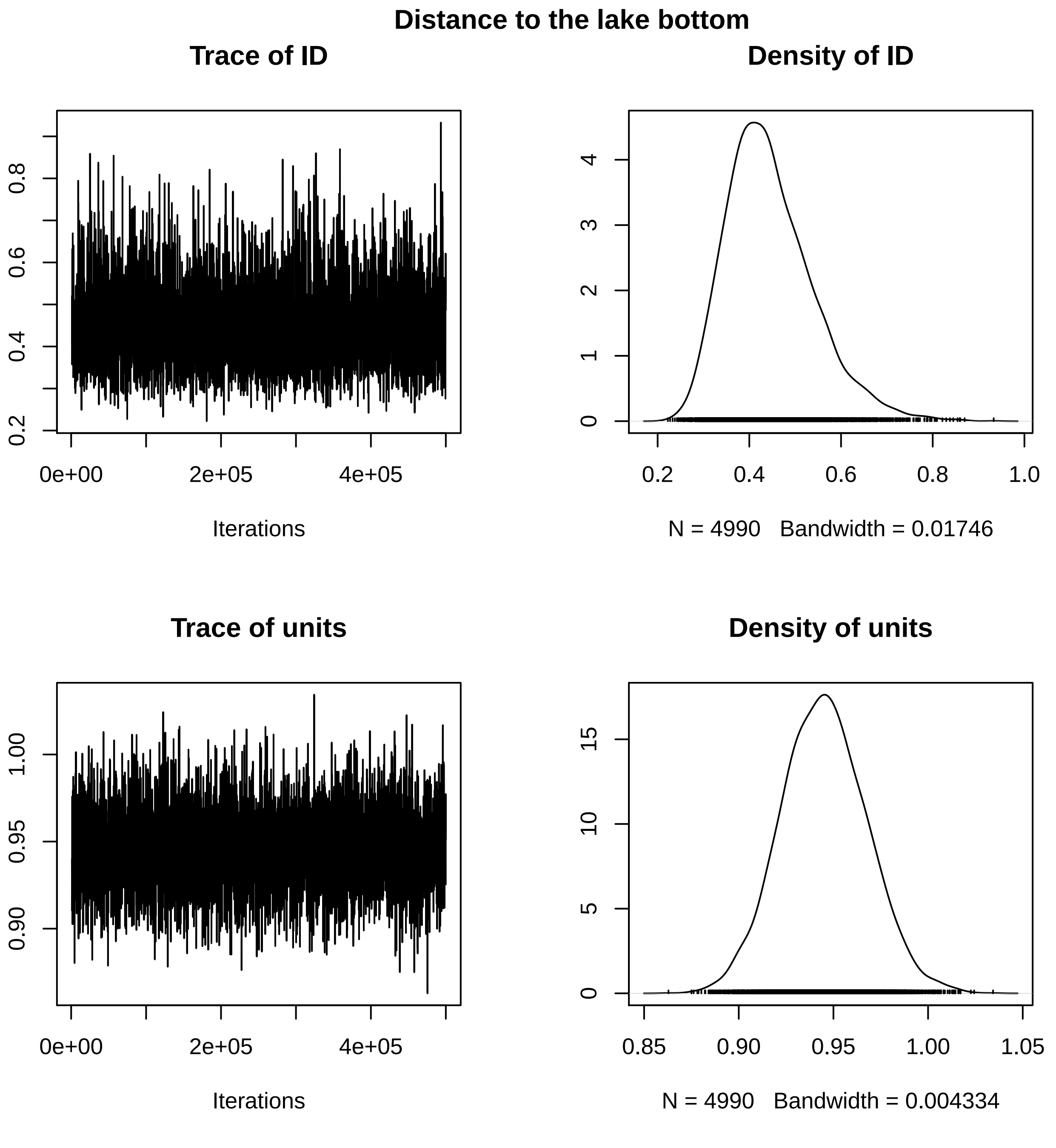
Distance to the lake bottom
Trace of ID
Density of ID
0.2
0.4
0.6
0.8
0e+00
2e+05
4e+05
Iterations
0
1
2
3
4
0.2
0.4
0.6
0.8
1.0
N = 4990   Bandwidth = 0.01746
Trace of units
Density of units
0.90
0.95
1.00
0e+00
2e+05
4e+05
Iterations
0
5
10
15
0.85
0.90
0.95
1.00
1.05
N = 4990   Bandwidth = 0.004334

Distance swam

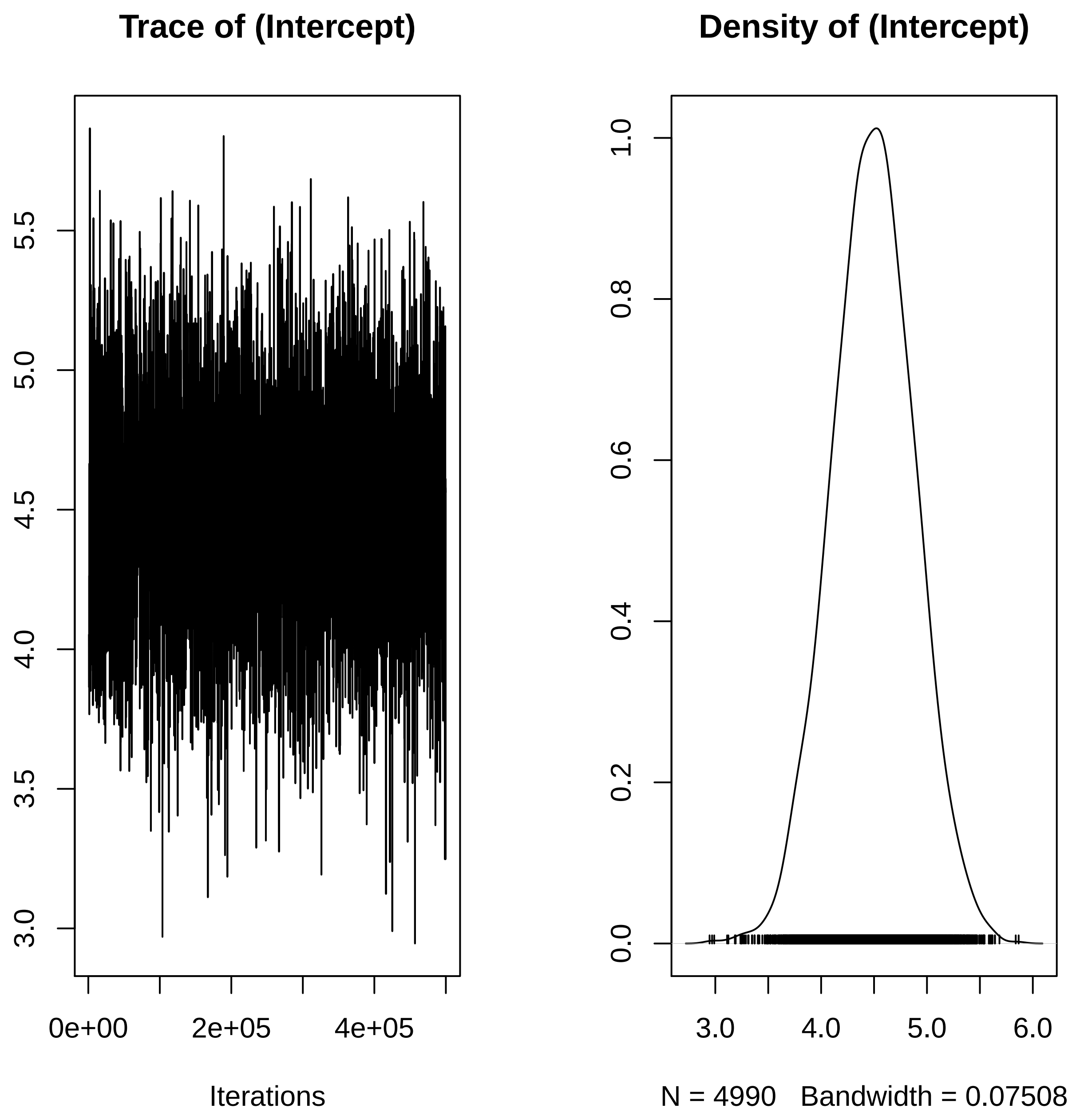

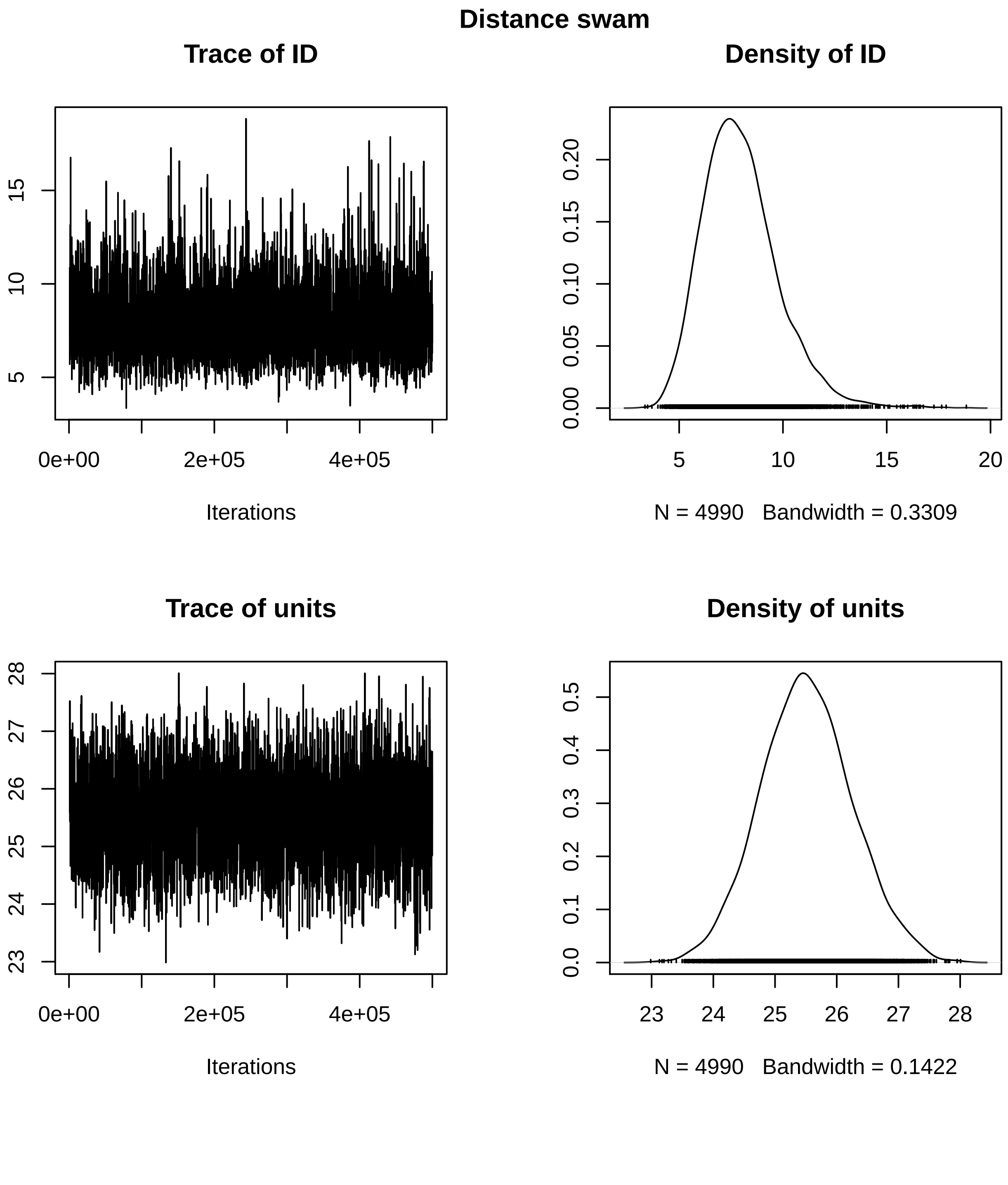
Distance swam
Trace of ID
Density of ID
15
10
5
0e+00
2e+05
4e+05
Iterations
0.20
0.15
0.10
0.05
0.00
5
10
15
20
N = 4990 Bandwidth = 0.3309
Trace of units
Density of units
28
27
26
25
24
23
0e+00
2e+05
4e+05
Iterations
0.5
0.4
0.3
0.2
0.1
0.0
23
24
25
26
27
28
N = 4990 Bandwidth = 0.1422

Time within 15 m of the feeding sites

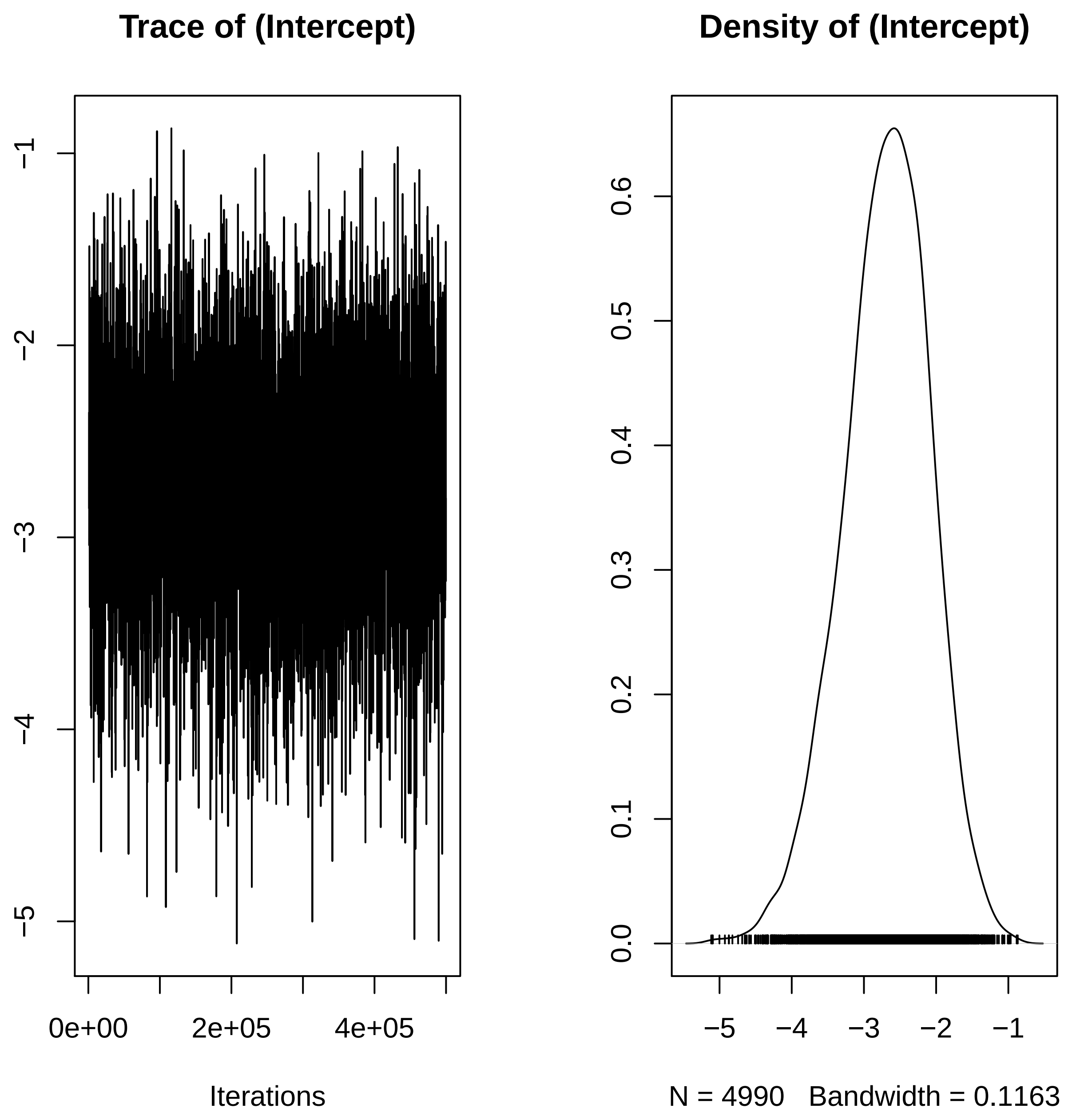

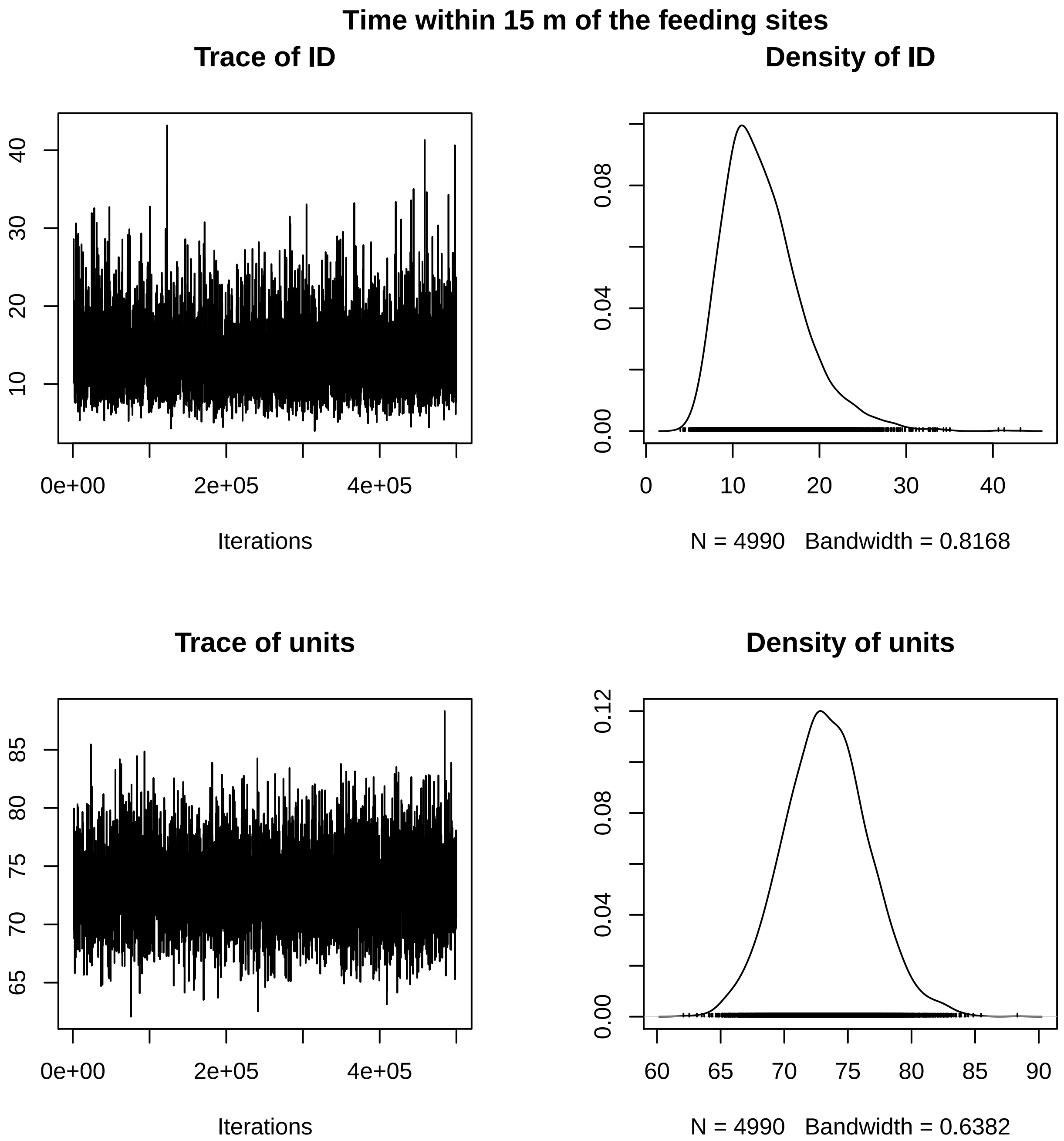
Time within 15 m of the feeding sites
Trace of ID
40
30
20
10
0e+00
2e+05
4e+05
Iterations
Density of ID
0.08
0.04
0.00
0
10
20
30
40
N = 4990 Bandwidth = 0.8168
Trace of units
85
80
75
70
65
0e+00
2e+05
4e+05
Iterations
Density of units
0.12
0.08
0.04
0.00
60
65
70
75
80
85
90
N = 4990 Bandwidth = 0.6382

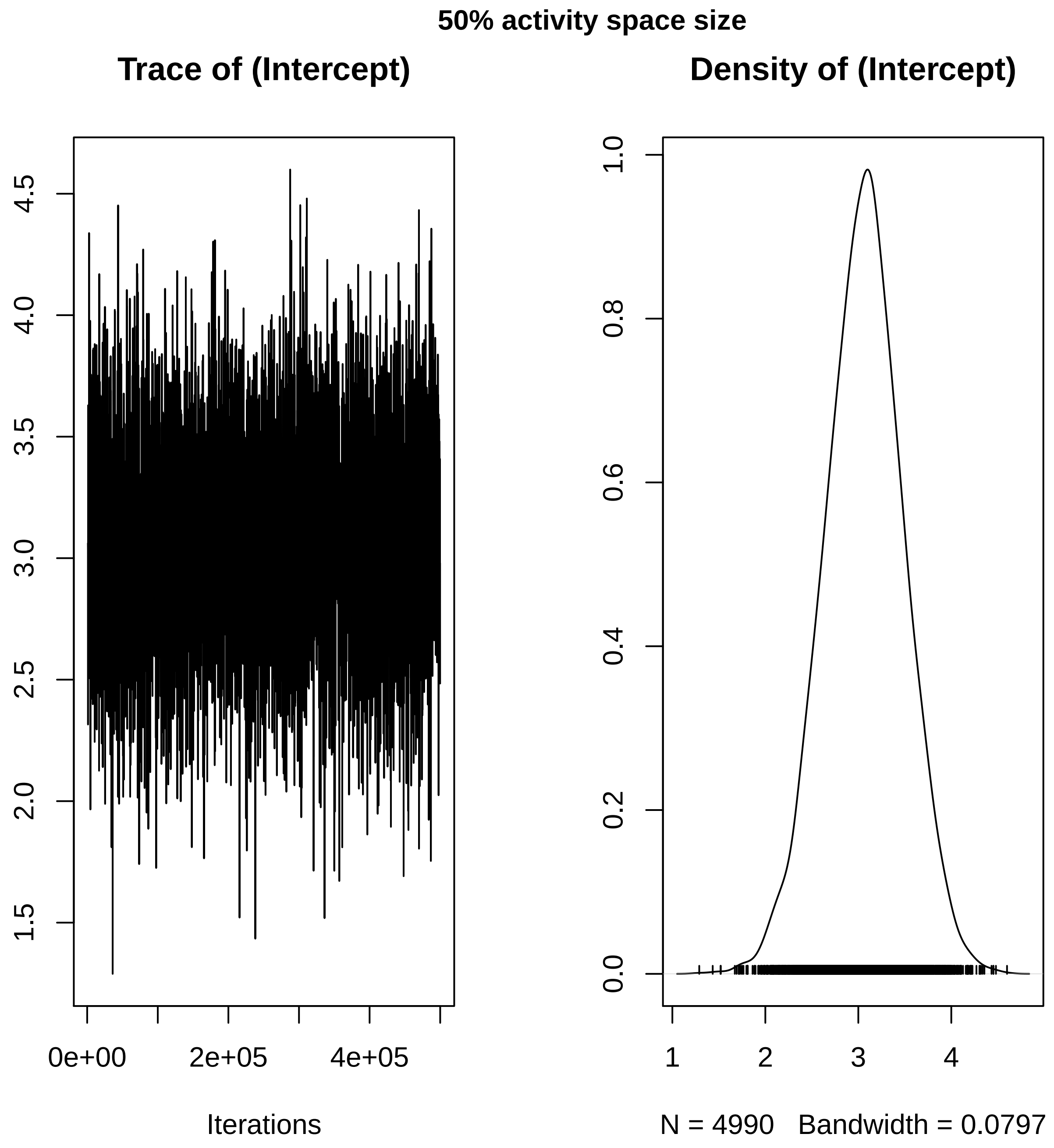
50% activity space size
Trace of (Intercept)
Density of (Intercept)
4.5
4.0
3.5
3.0
2.5
2.0
1.5
0e+00
2e+05
4e+05
Iterations
1.0
0.8
0.6
0.4
0.2
0.0
1
2
3
4
N = 4990 Bandwidth = 0.0797

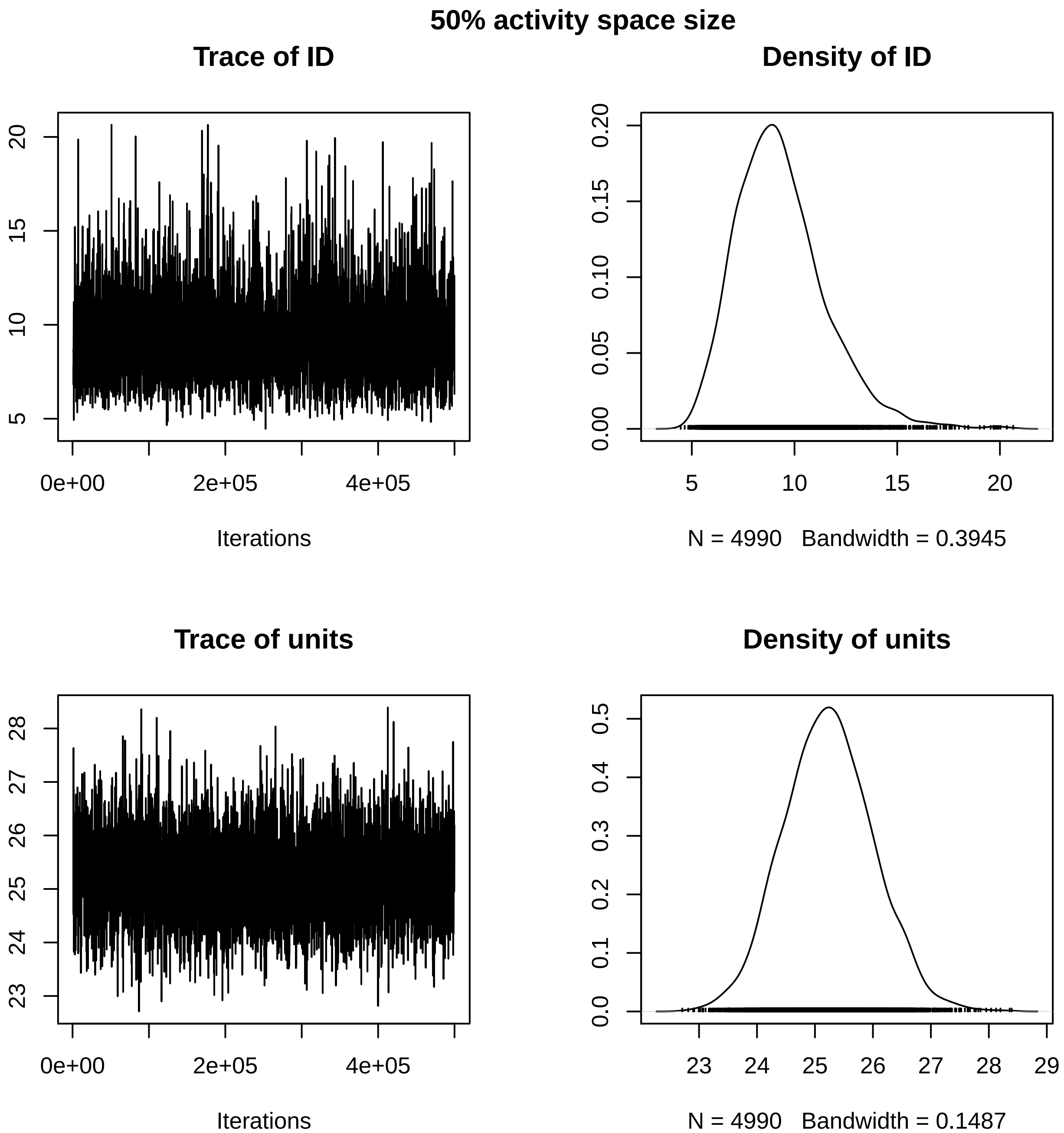
50% activity space size
Trace of ID
Density of ID
Iterations
N = 4990 Bandwidth = 0.3945
Trace of units
Density of units
Iterations
N = 4990 Bandwidth = 0.1487

Number of switches among feeding sites

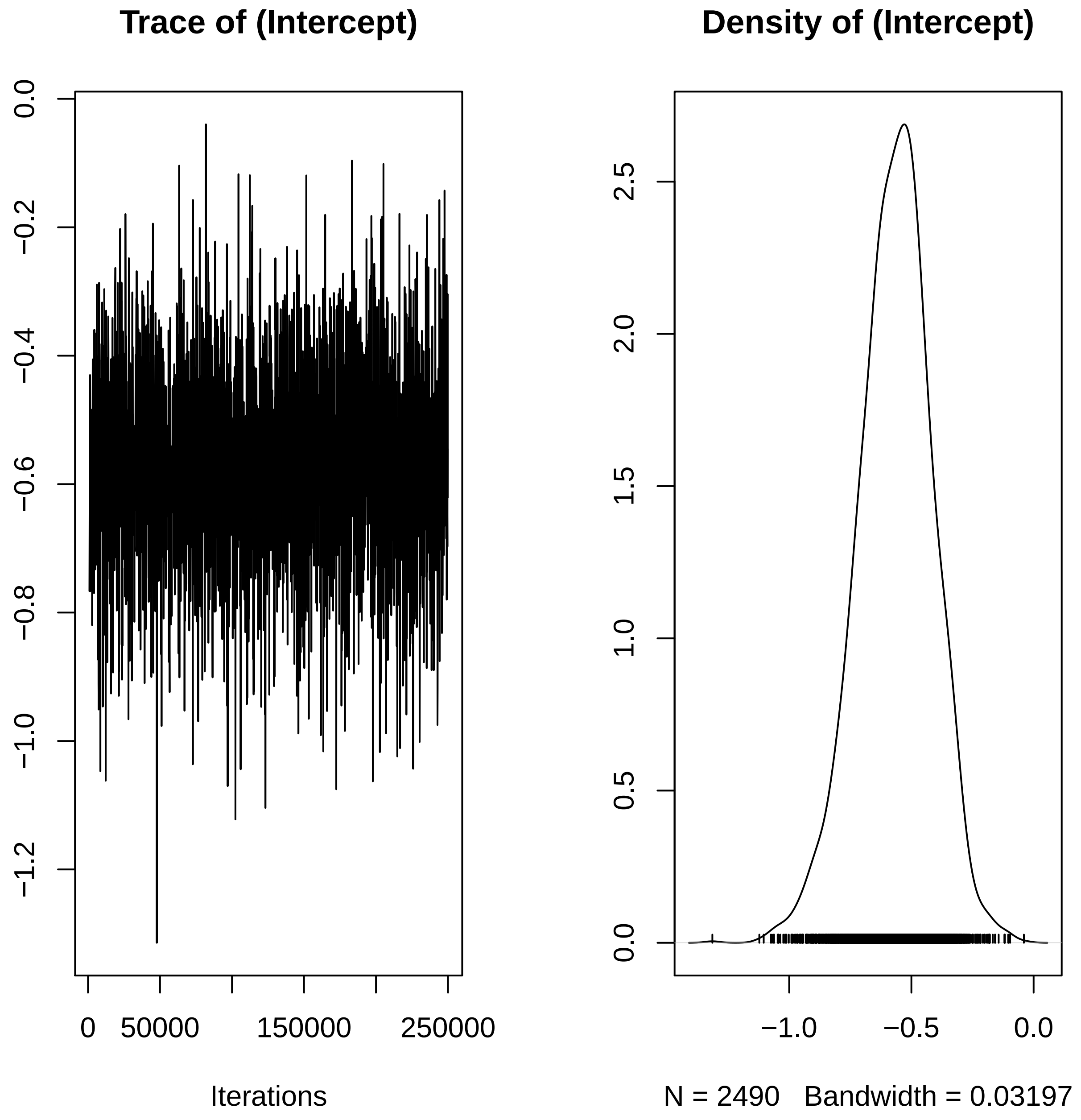

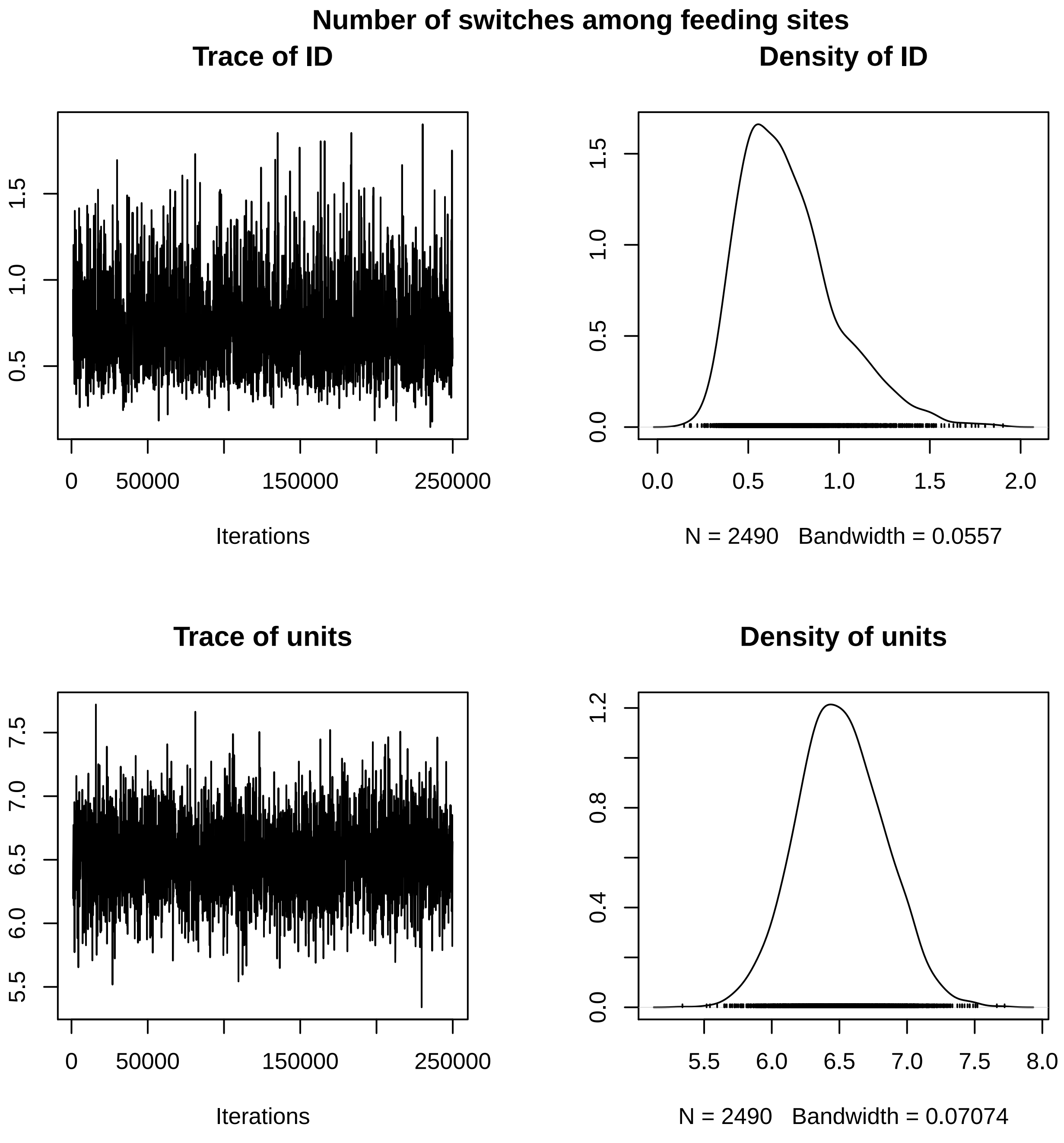
Number of switches among feeding sites
Trace of ID
Density of ID
1.5
1.0
0.5
0 50000 150000 250000
Iterations
0.0 0.5 1.0 1.5 2.0
N = 2490 Bandwidth = 0.0557
Trace of units
Density of units
7.5
7.0
6.5
6.0
5.5
1.2
0.8
0.4
0.0
5.5 6.0 6.5 7.0 7.5 8.0
N = 2490 Bandwidth = 0.07074

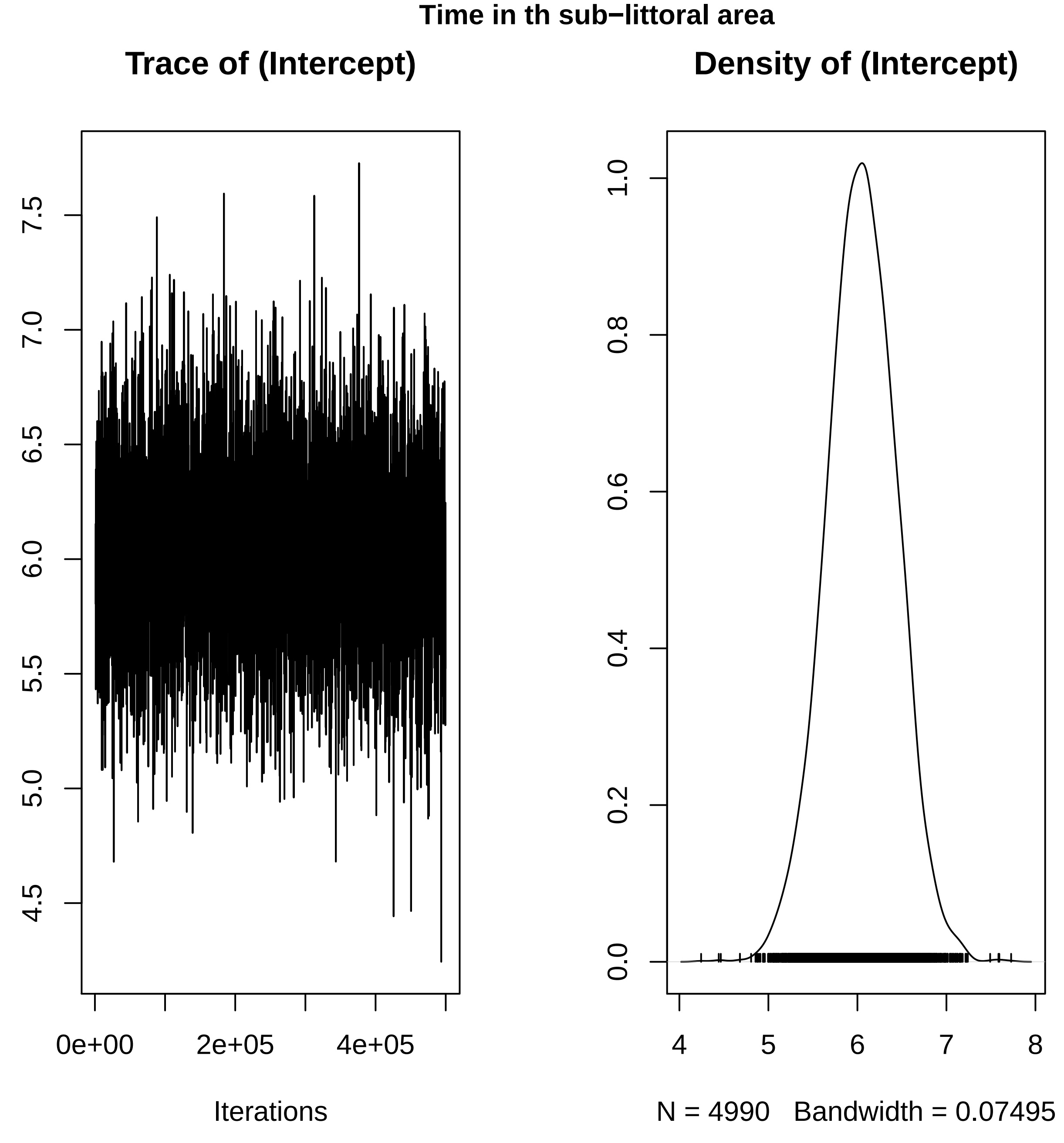

Time in th sub−littoral area
Trace of (Intercept)
Density of (Intercept)
7.5
7.0
6.5
6.0
5.5
5.0
4.5
0e+00
2e+05
4e+05
Iterations
1.0
0.8
0.6
0.4
0.2
0.0
4
5
6
7
8
N = 4990   Bandwidth = 0.07495

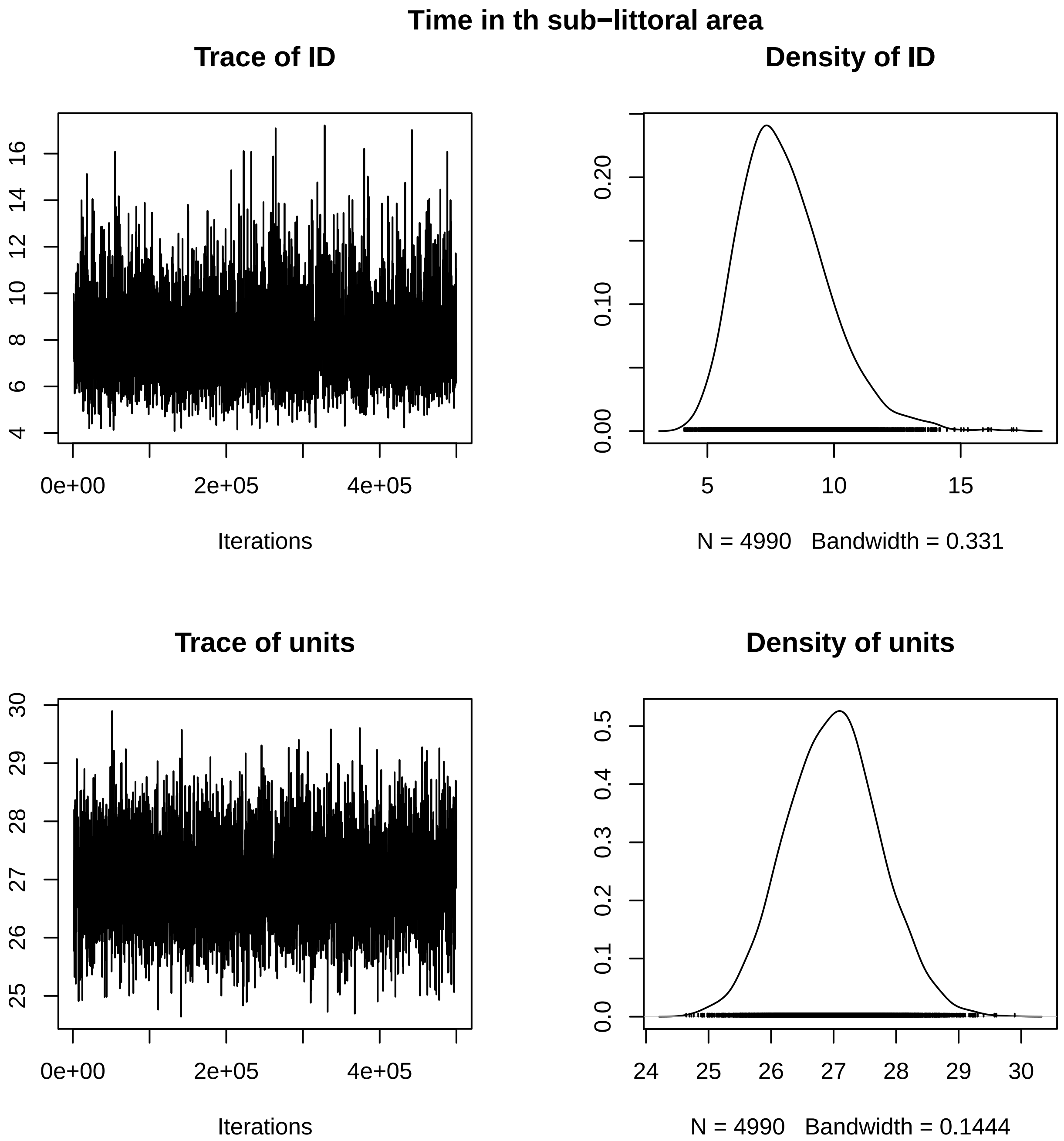
Time in th sub−littoral area
Trace of ID
Density of ID
Iterations
N = 4990 Bandwidth = 0.331
Trace of units
Density of units
Iterations
N = 4990 Bandwidth = 0.1444
