# Supplementary material for: Encountering a bait is necessary but insufficient to explain individual variability in vulnerability to angling in two freshwater benthivorous fish in the wild
Source: PLoS One. 2017 Mar 16;12(3):e0173989. doi: 10.1371/journal.pone.0173989 (PMC5354434; doi:10.1371/journal.pone.0173989)
Supplement: S3 Appendix — (PDF) [file pone.0173989.s003.pdf]

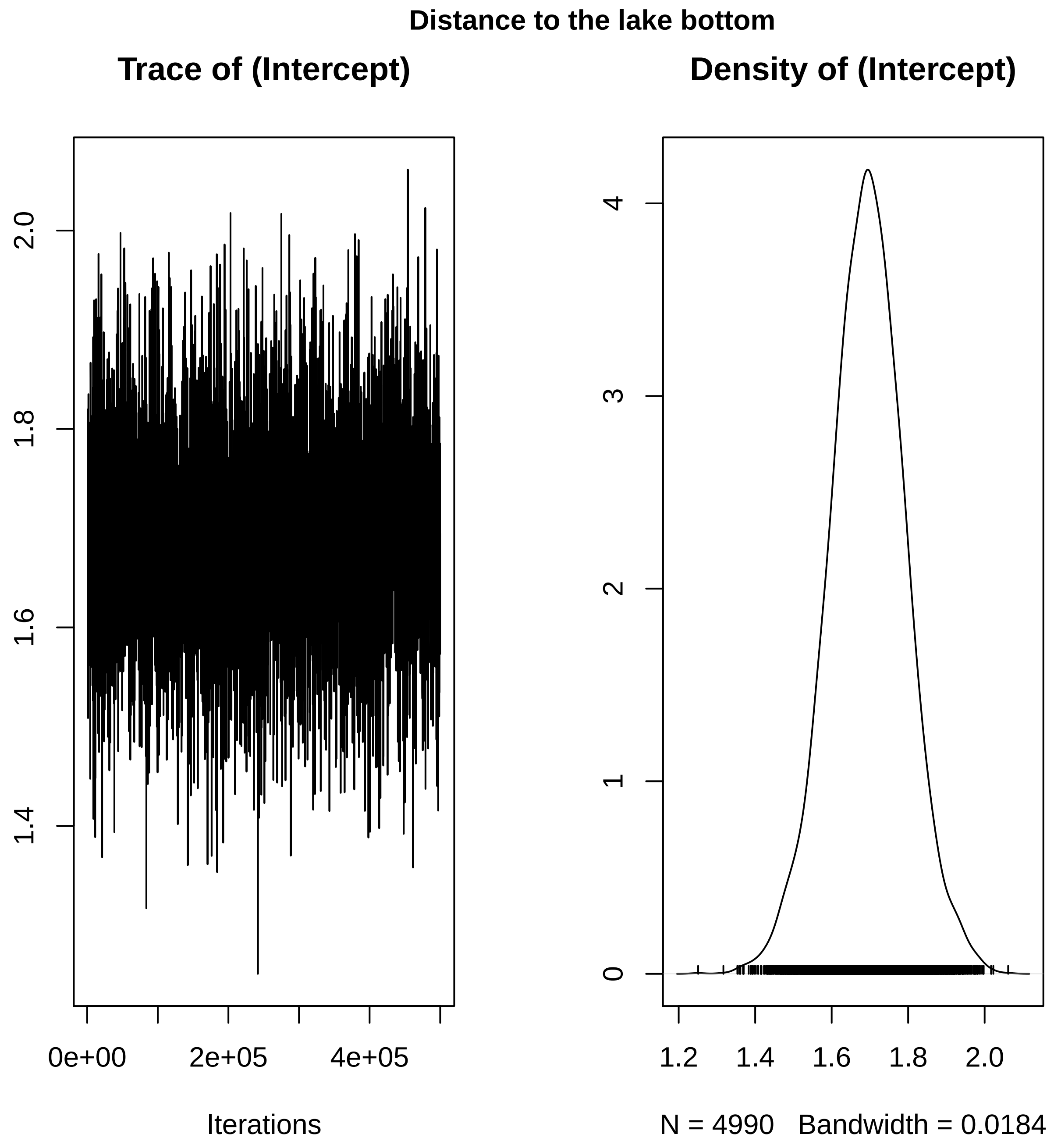
Distance to the lake bottom
Trace of (Intercept)
Density of (Intercept)
2.0
1.8
1.6
1.4
0e+00
2e+05
4e+05
Iterations
4
3
2
1
0
1.2
1.4
1.6
1.8
2.0
N = 4990 Bandwidth = 0.0184

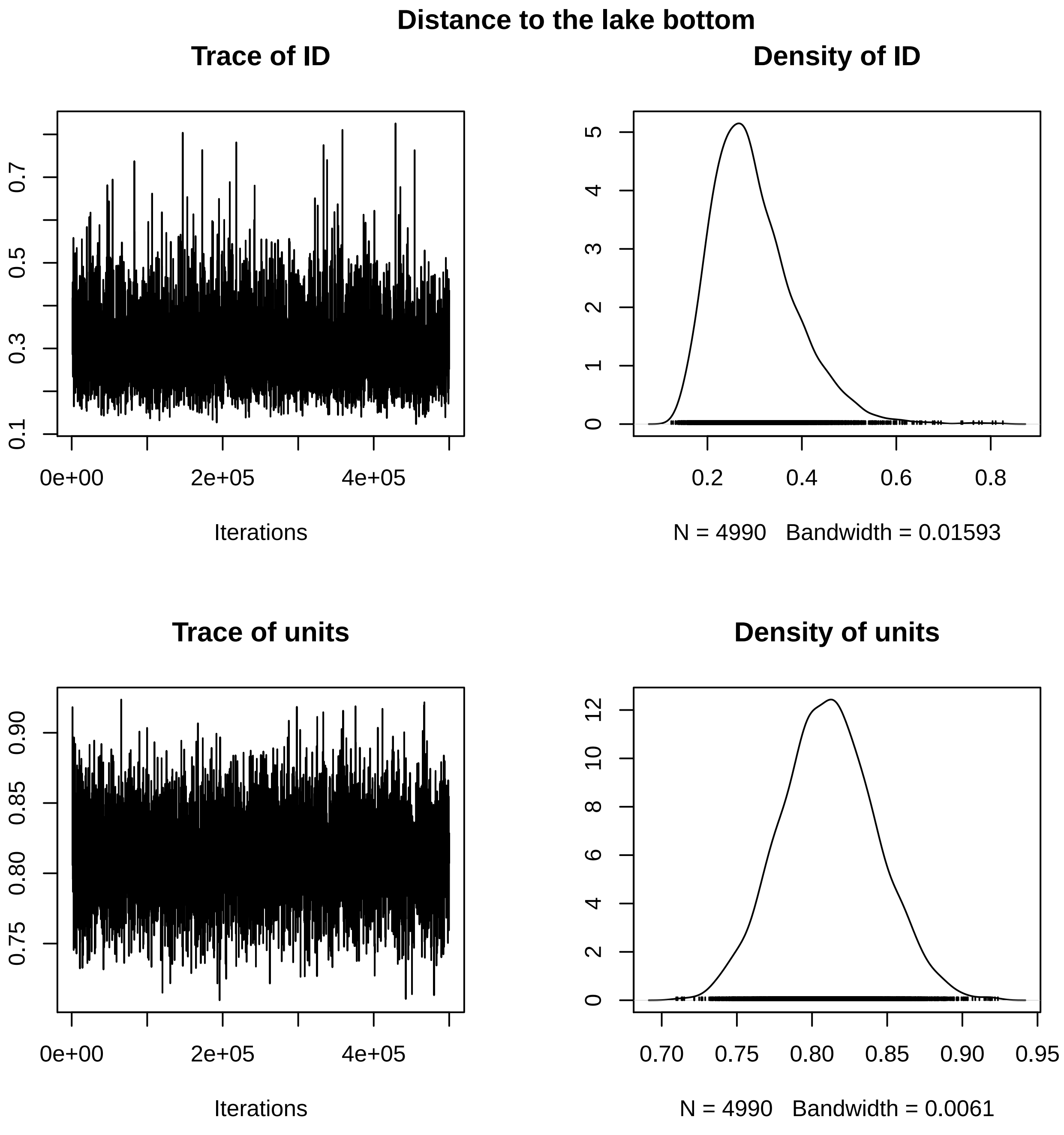
Distance to the lake bottom
Trace of ID
Density of ID
0.1
0.3
0.5
0.7
0e+00
2e+05
4e+05
Iterations
0
1
2
3
4
5
0.2
0.4
0.6
0.8
N = 4990 Bandwidth = 0.01593
Trace of units
Density of units
0.75
0.80
0.85
0.90
0e+00
2e+05
4e+05
Iterations
0
2
4
6
8
10
12
0.70
0.75
0.80
0.85
0.90
0.95
N = 4990 Bandwidth = 0.0061

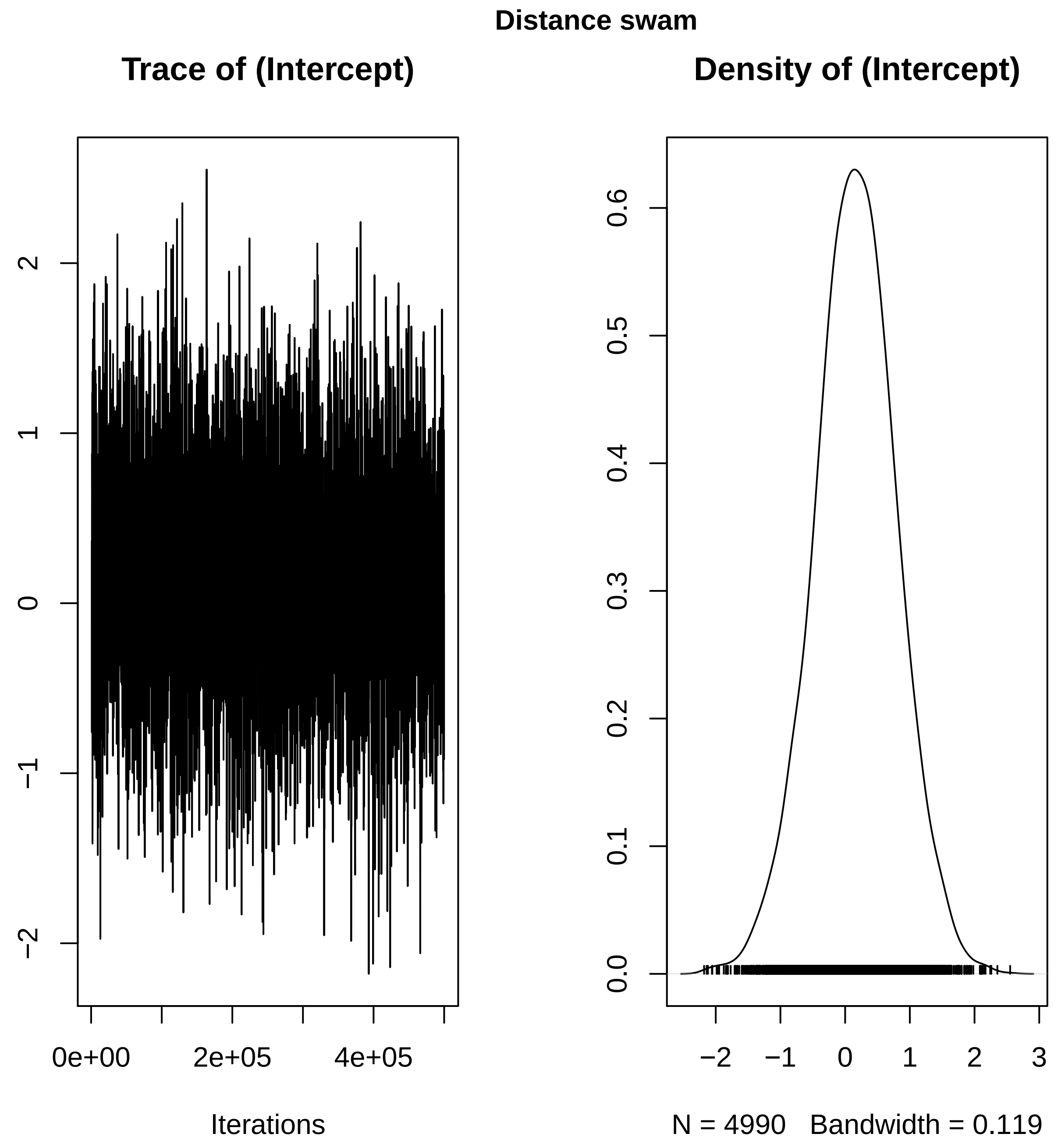
Distance swam
Trace of (Intercept)
Density of (Intercept)
−2
−1
0
1
2
0e+00
2e+05
4e+05
Iterations
0.0
0.1
0.2
0.3
0.4
0.5
0.6
−2
−1
0
1
2
3
N = 4990 Bandwidth = 0.119

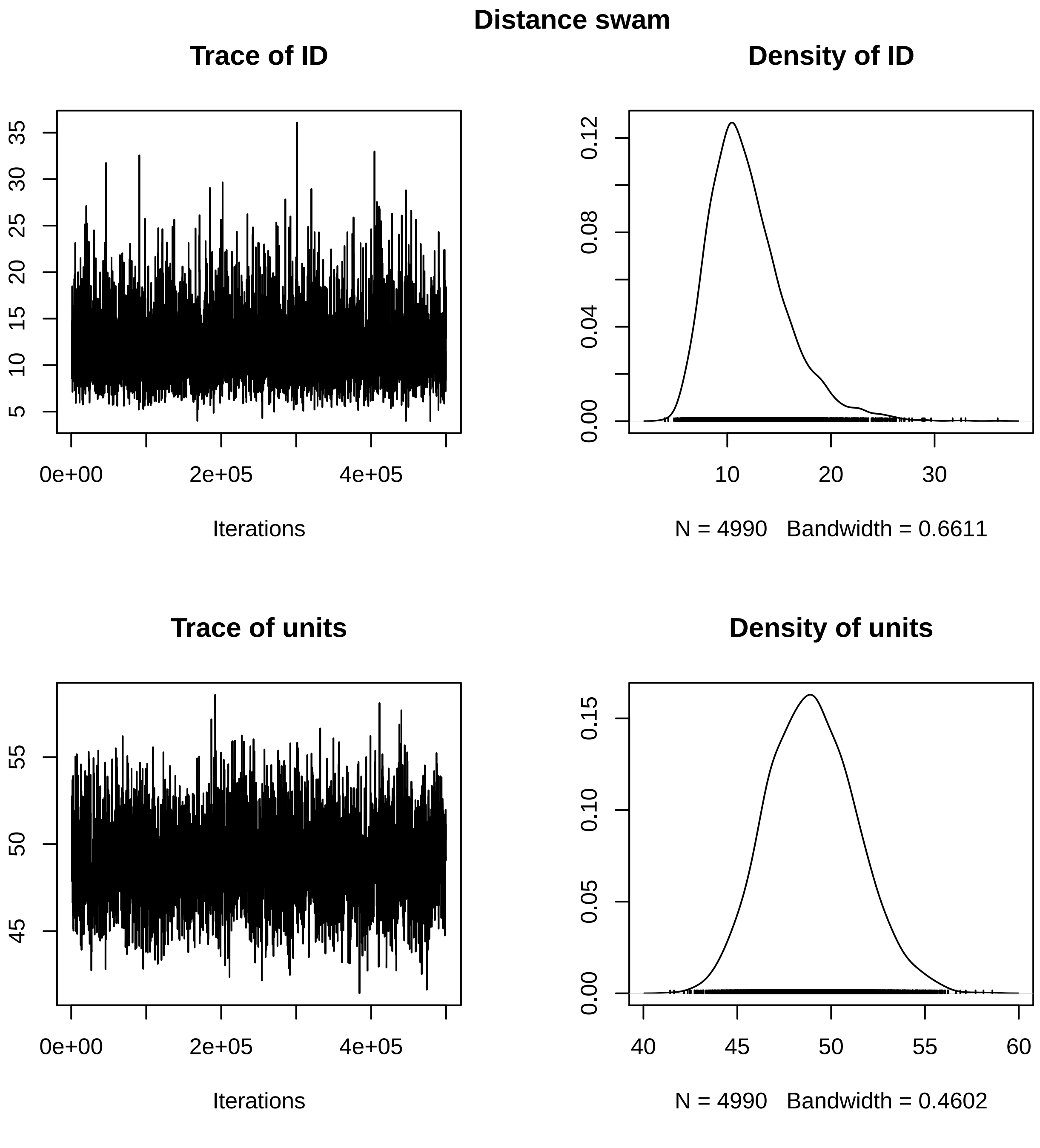
Distance swam
Trace of ID
Density of ID
Iterations
N = 4990 Bandwidth = 0.6611
Trace of units
Density of units
Iterations
N = 4990 Bandwidth = 0.4602

Time within 15 m of the feeding sites

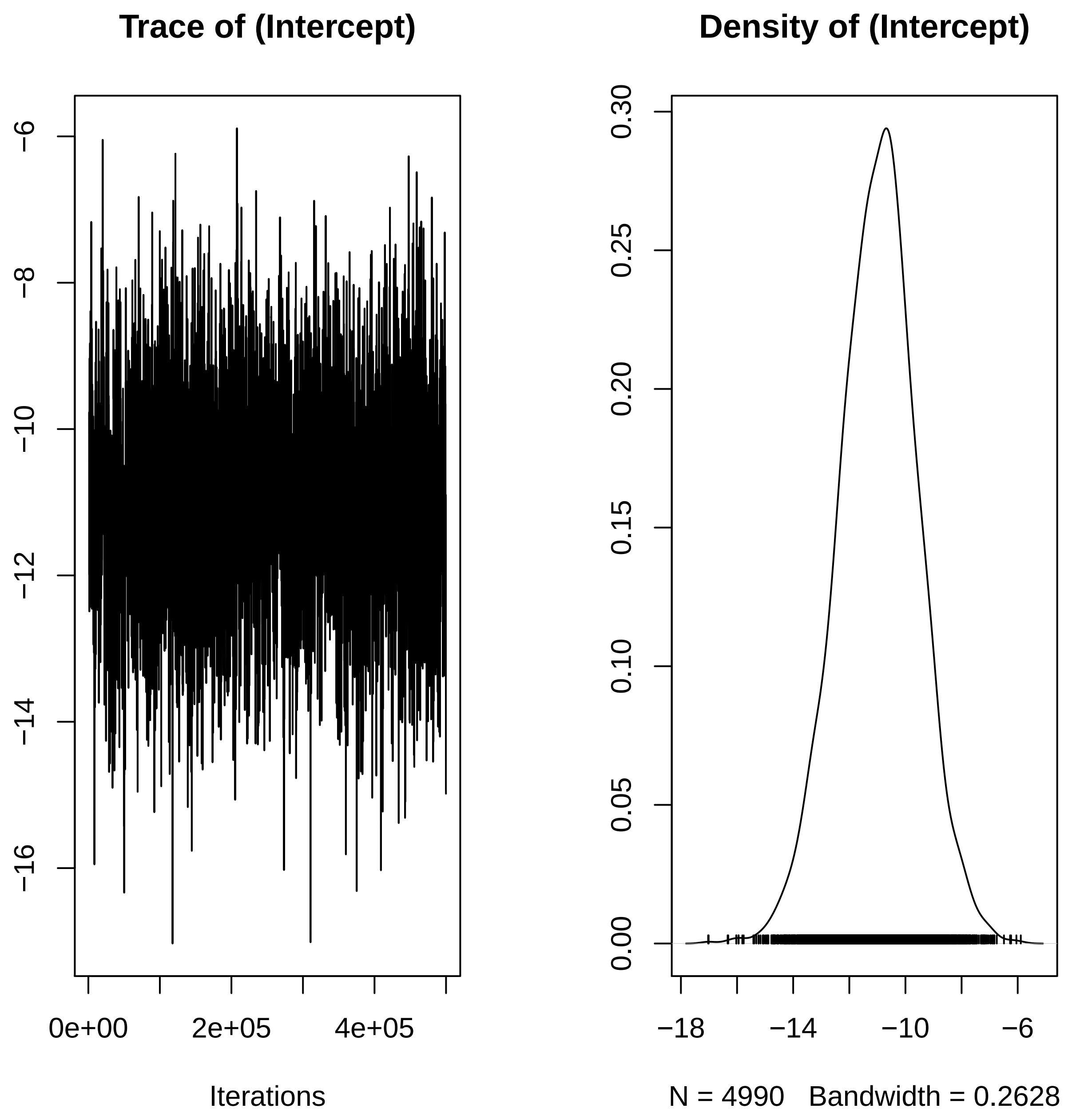

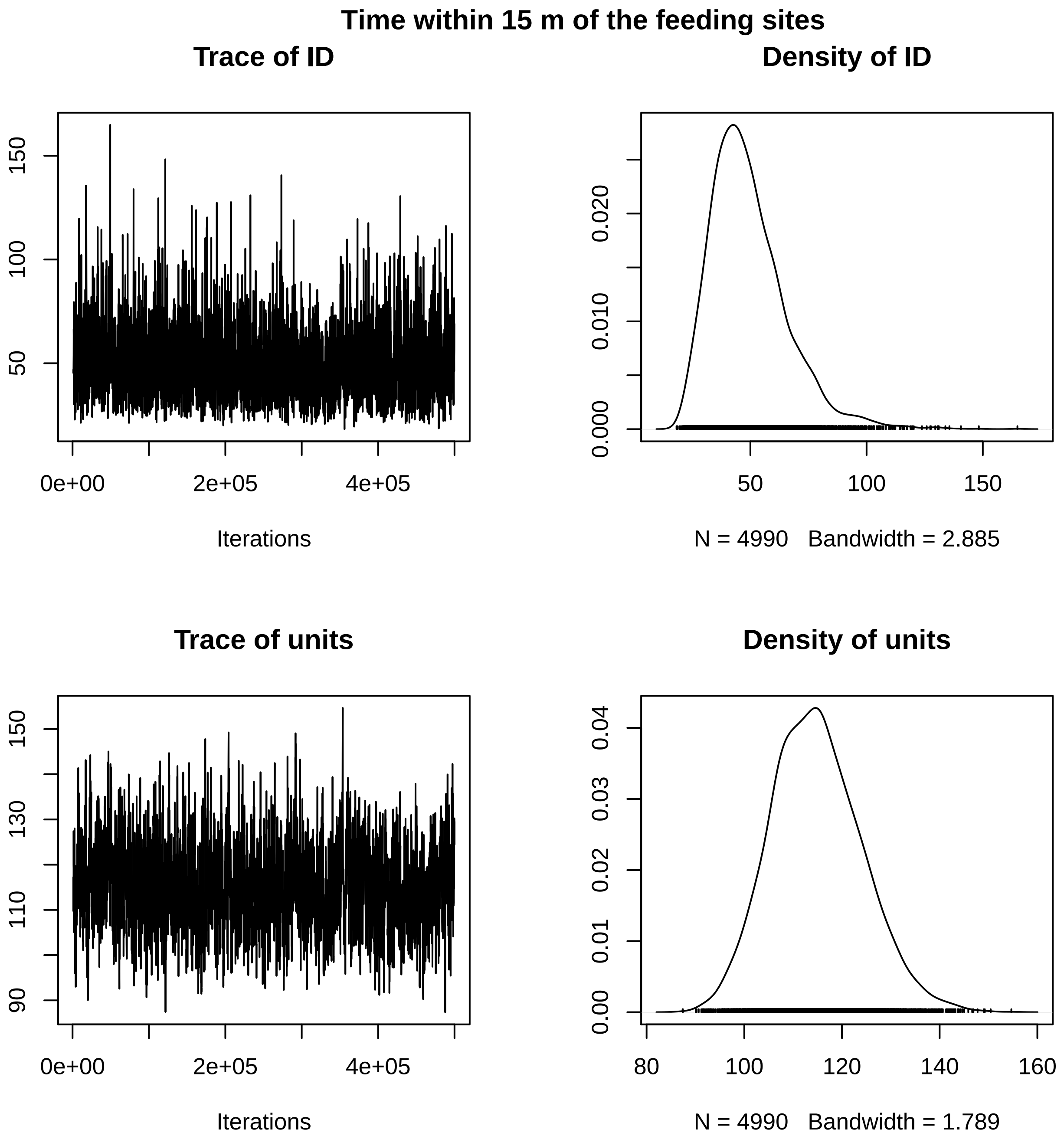

Time within 15 m of the feeding sites
Trace of ID
Density of ID
Iterations
N = 4990 Bandwidth = 2.885
Trace of units
Density of units
Iterations
N = 4990 Bandwidth = 1.789

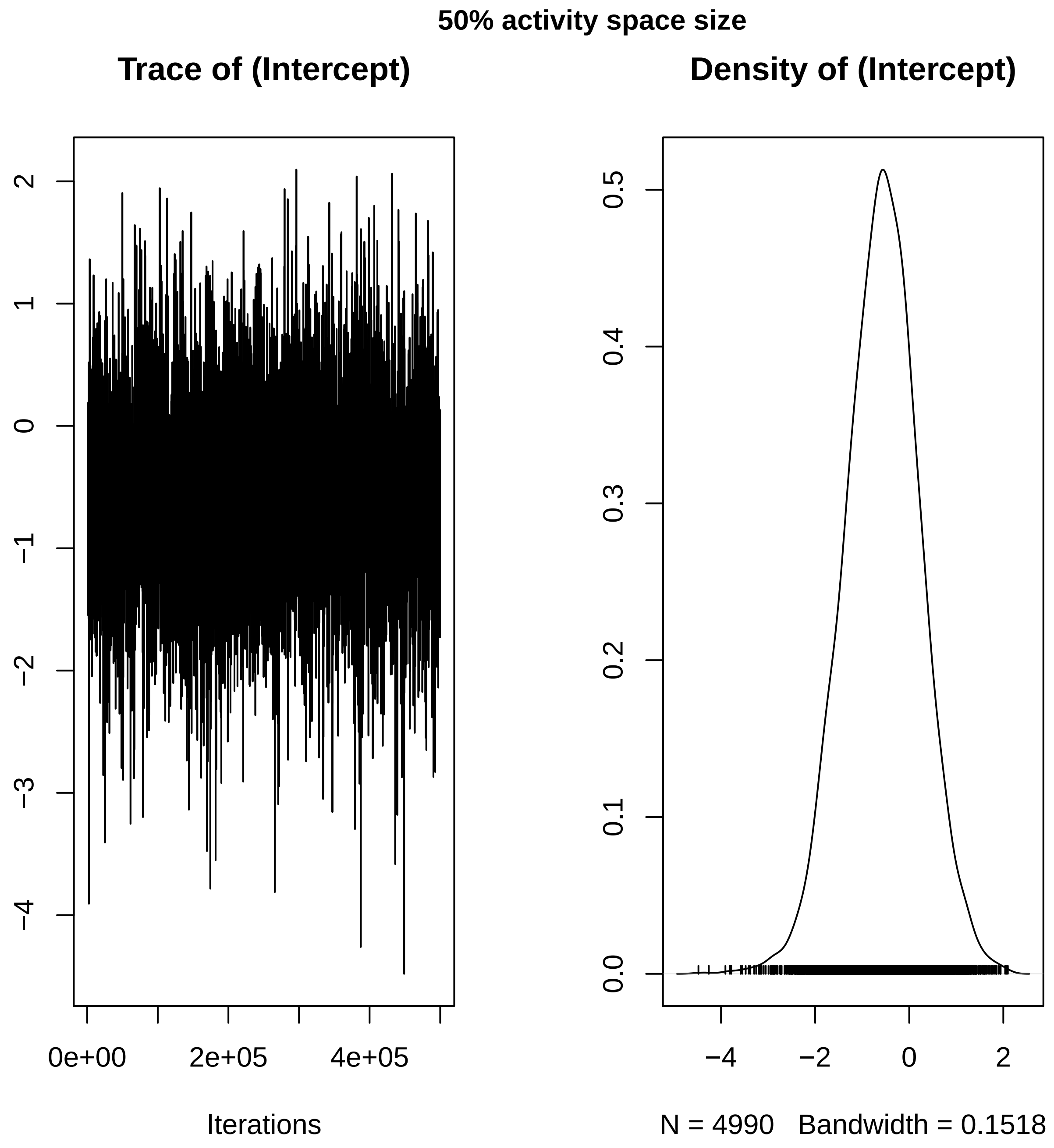
50% activity space size
Trace of (Intercept)
Density of (Intercept)
Iterations
N = 4990 Bandwidth = 0.1518
0e+00
2e+05
4e+05

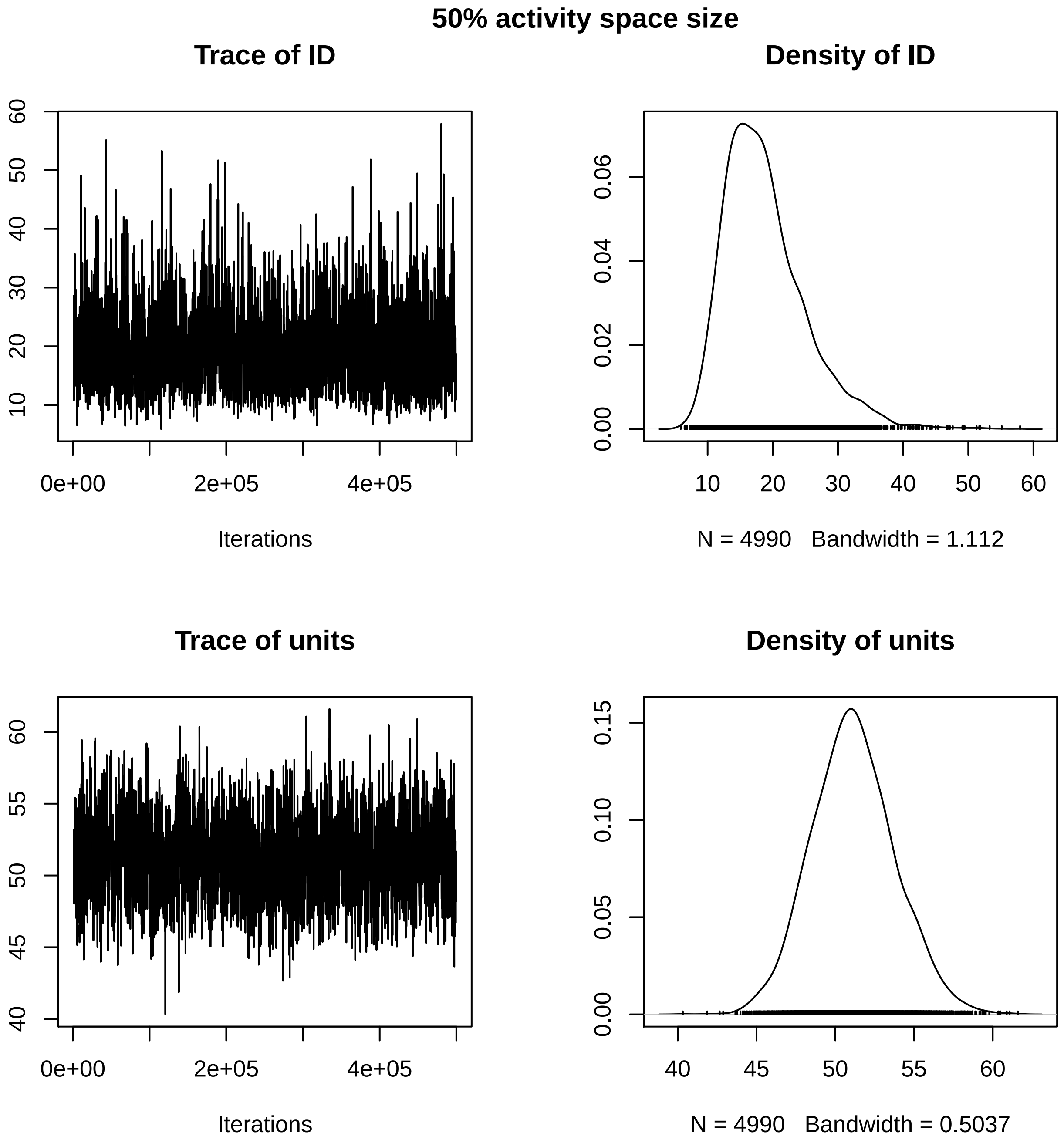
50% activity space size
Trace of ID
Iterations
Density of ID
N = 4990 Bandwidth = 1.112
Trace of units
Iterations
Density of units
N = 4990 Bandwidth = 0.5037

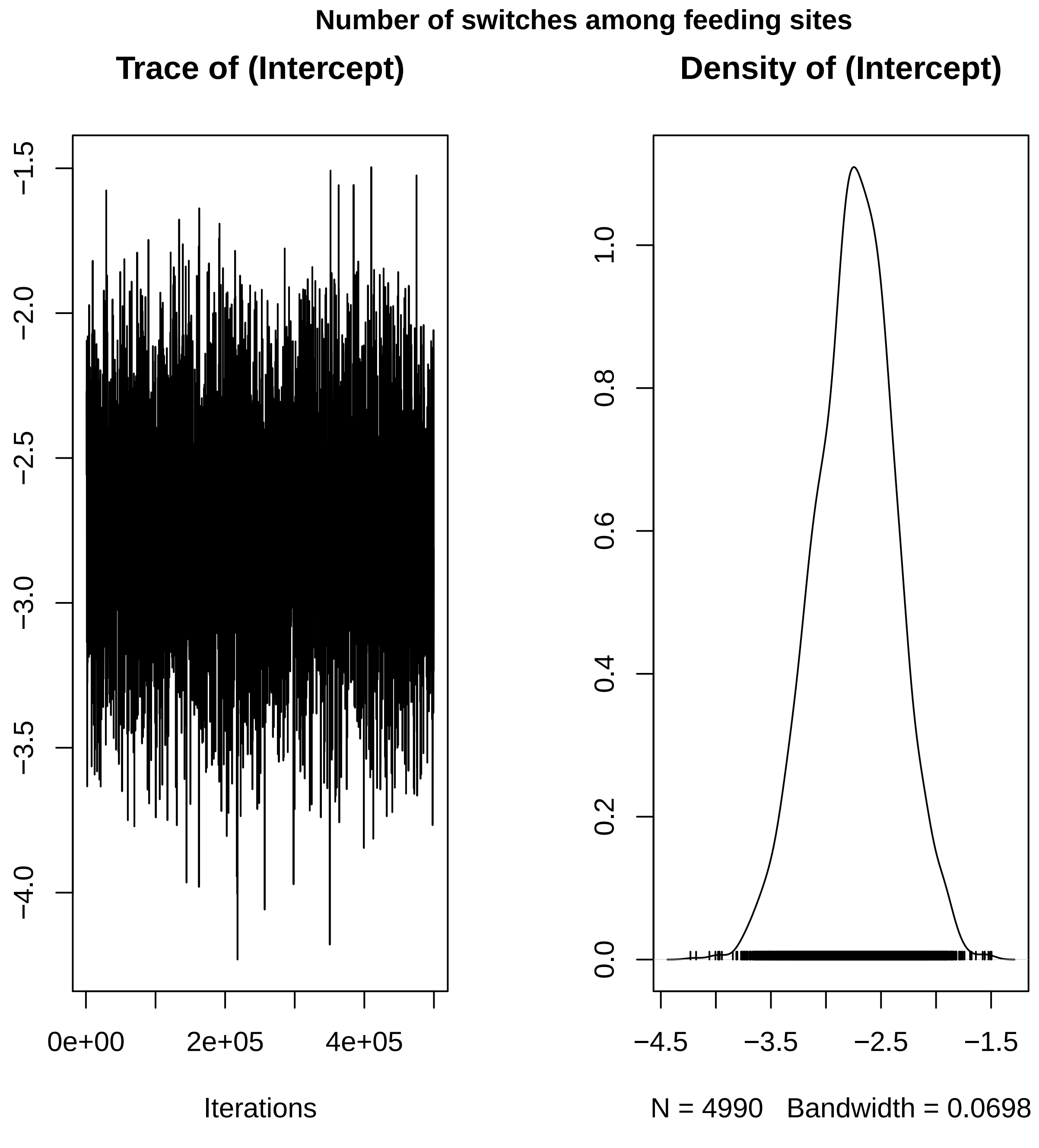

Number of switches among feeding sites
Trace of (Intercept)
Density of (Intercept)
−1.5
−2.0
−2.5
−3.0
−3.5
−4.0
0e+00
2e+05
4e+05
Iterations
1.0
0.8
0.6
0.4
0.2
0.0
−4.5
−3.5
−2.5
−1.5
N = 4990 Bandwidth = 0.0698

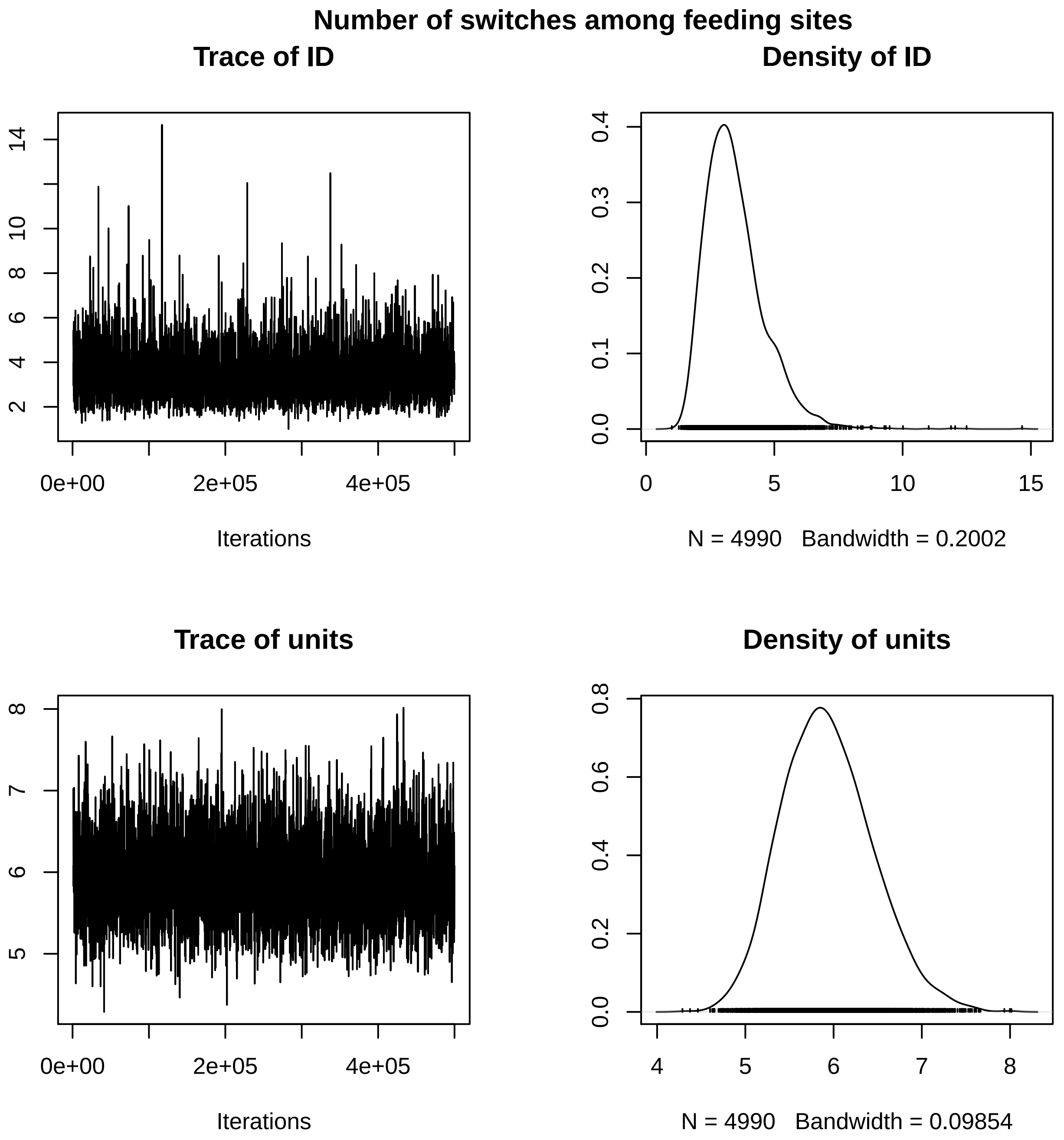
Number of switches among feeding sites
Trace of ID
Density of ID
Iterations
N = 4990   Bandwidth = 0.2002
Trace of units
Density of units
Iterations
N = 4990   Bandwidth = 0.09854
0e+00
2e+05
4e+05

Time in th sub−littoral area

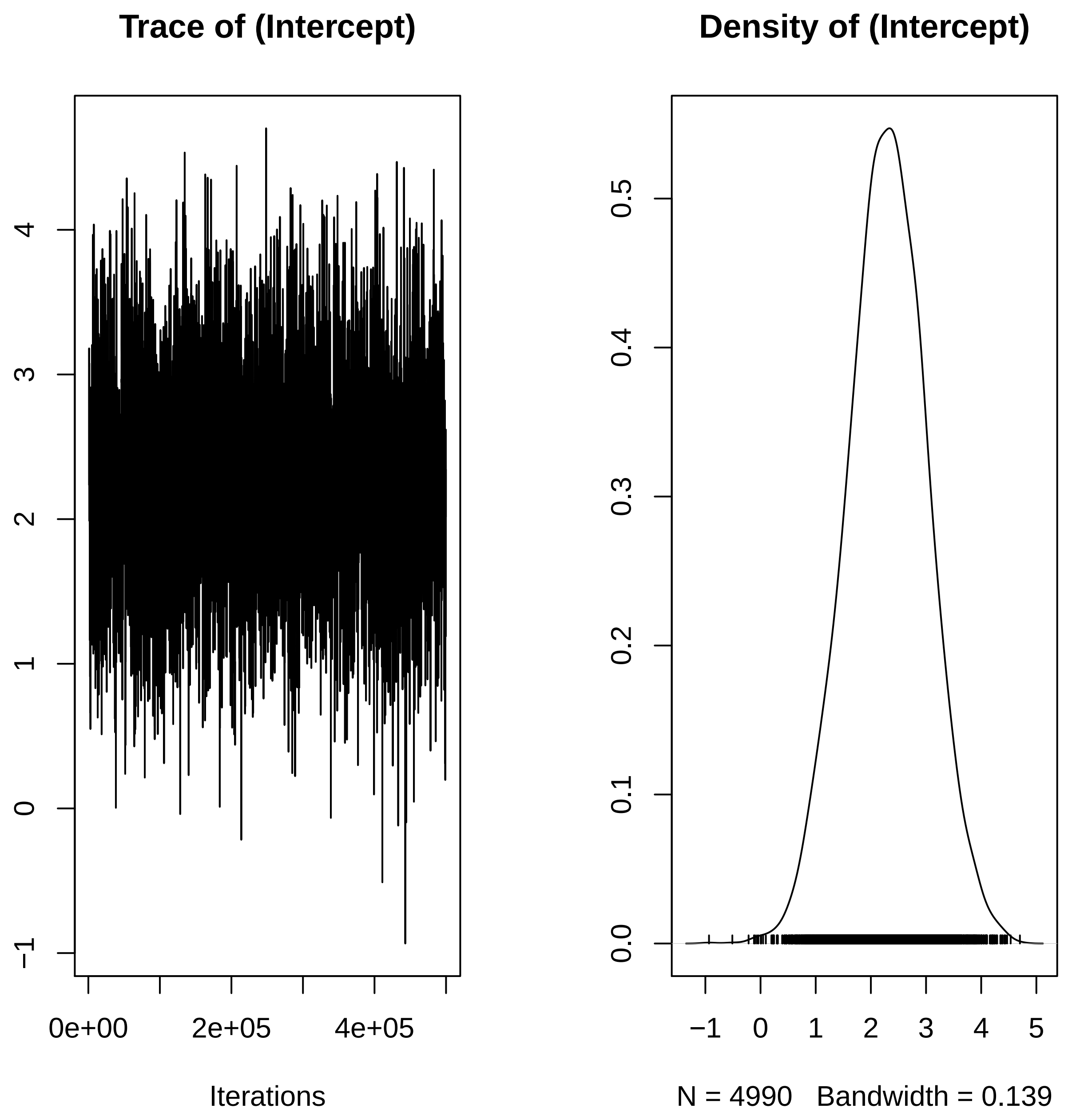

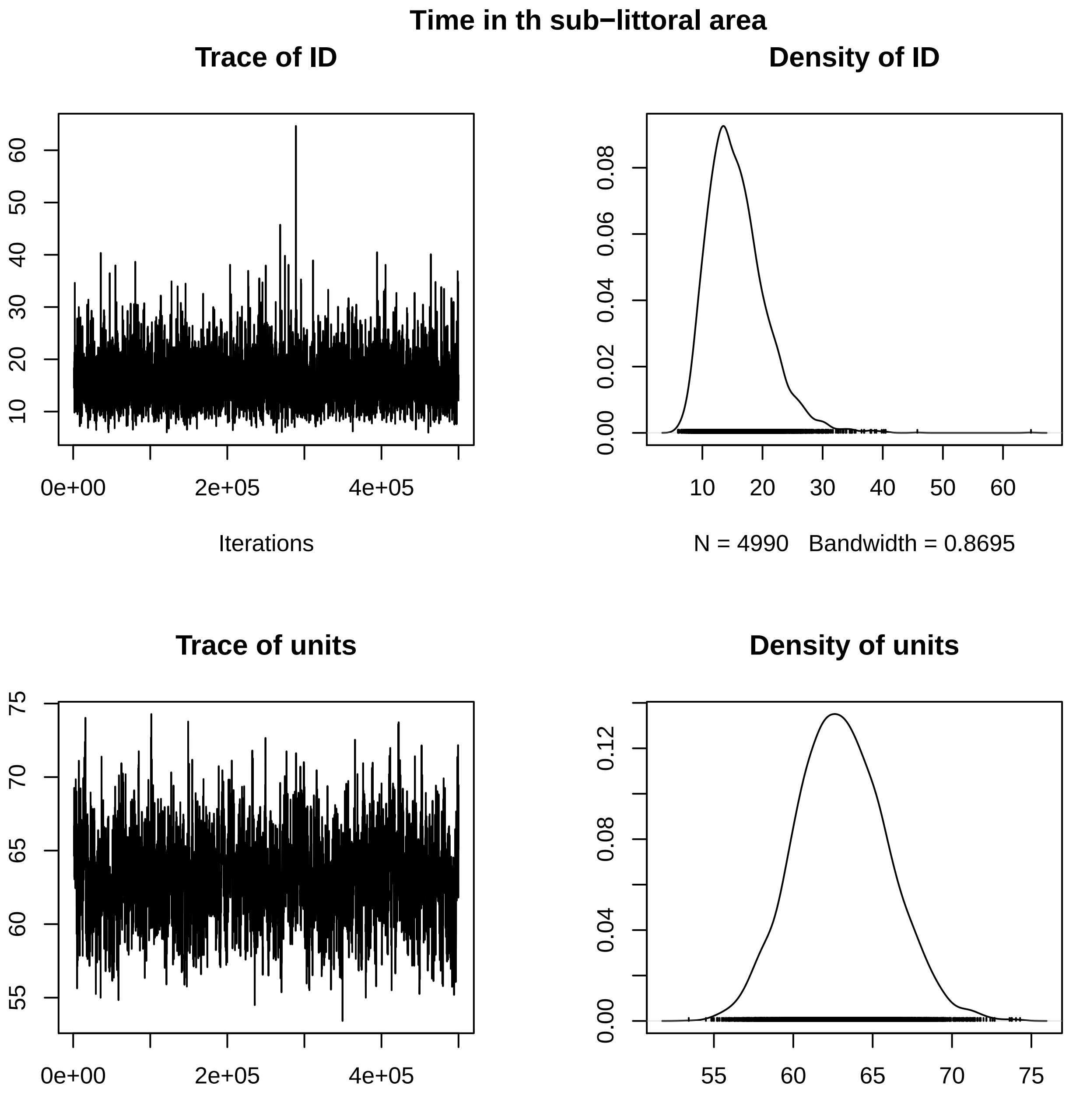
Time in th sub−littoral area
Trace of ID
Density of ID
60
50
40
30
20
10
0e+00
2e+05
4e+05
Iterations
0.08
0.06
0.04
0.02
0.00
10
20
30
40
50
60
N = 4990 Bandwidth = 0.8695
Trace of units
Density of units
75
70
65
60
55
0e+00
2e+05
4e+05
Iterations
0.12
0.08
0.04
0.00
55
60
65
70
75
N = 4990 Bandwidth = 0.5585
